# Cotton cytochrome P450 CYP82D regulates systemic cell death by modulating the octadecanoid pathway

**DOI:** 10.1038/ncomms6372

**Published:** 2014-11-05

**Authors:** Longqing Sun, Longfu Zhu, Li Xu, Daojun Yuan, Ling Min, Xianlong Zhang

**Affiliations:** 1 National Key Laboratory of Crop Genetic Improvement, Huazhong Agricultural University, Wuhan, Hubei 430070, China

## Abstract

Plant oxylipins are derived from unsaturated fatty acids and play roles in plant growth and development as well as defence. Although recent studies have revealed that fatty acid metabolism is involved in systemic acquired resistance, the precise function of oxylipins in plant defence remains unknown. Here we report a cotton P450 gene *SILENCE-INDUCED STEM NECROSIS* (*SSN*), RNAi suppression of which causes a lesion mimic phenotype. SSN is also involved in jasmonate metabolism and the response to wounding. Fatty acid and oxylipin metabolite analysis showed that *SSN* overexpression causes hyperaccumulation of hydroxide and ketodiene fatty acids and reduced levels of 18:2 fatty acids, whereas silencing causes an imbalance in *LOX* (lipoxygenase) expression and excessive hydroperoxide fatty acid accumulation. We also show that an unknown oxylipin-derived factor is a putative mobile signal required for systemic cell death and hypothesize that SSN acts as a valve to regulate HR on pathogen infection.

## Abstract (web summary)

Oxylipin signalling is known to play important roles in plant growth, development and defence against pathogens. Here Sun *et al.* identify a novel cytochrome P450 in cotton and show that its suppression leads to activation of plant defence responses and alteration of oxylipin metabolism.

## Graphical abstract

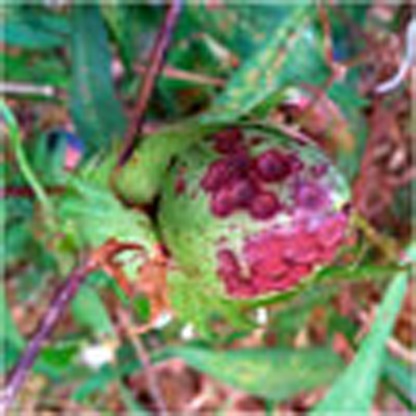

Plants are constantly challenged by a barrage of microbes, but only a small proportion succeed in causing disease due to the well-established immune system of plants. Plant innate immune systems comprise complex signalling networks that generally include two modes, pathogen-associated molecular pattern-triggered immunity (PTI) and effector-triggered immunity (ETI)^1^. PTI is induced when pathogen-associated molecular patterns, which are conserved molecules such as flagellin, are perceived by extracellular receptors, the so-called pattern recognition receptors, such as FLS2 (ref. 2). However, when PTI is suppressed by pathogen effectors, and they are transported into the cell, plants can re-establish pathogen resistance using other defence modes, such as ETI^3^. ETI is induced when the avirulence effectors produced by a pathogen are recognized by the corresponding plant resistance proteins^4^. ETI is often accompanied by the hypersensitive response (HR), which includes an oxidative burst, cell wall lignification, phytoalexin accumulation and induction of cell death of infected cells and the cells that surround them, to prevent the pathogen from spreading^5^,^6^. HR is a form of programmed cell death (PCD) in plants^7^, which results in necrotic lesion formation, sealing the pathogen in a tomb of dead cells. This process is also associated with salicylic acid (SA) accumulation, which induces the expression of *PATHOGENESIS-RELATED* (*PR*) genes and eventually establishes systemic acquired resistance (SAR)^8^,^9^. Interestingly, SAR can be triggered in tobacco wild-type (WT) scion grafted onto an SA-deficient rootstock^10^. SAR has also been found to be blocked when SA methyl transferase was silenced in primary infected leaves, and SAR was induced in upper untreated leaves when lower leaves were treated with MeSA. Therefore, MeSA is a proposed SAR signal in tobacco^11^. Jasmonate (JA) rapidly accumulates in the phloem and exudates as the leaves are challenged with the avirulent *Pseudomonas syringae*; further, SAR can be mimicked by a foliar JA application and is abrogated in mutants with impaired JA synthesis or response^12^. In addition, the JA precursor 12-oxo-phytodienoic acid accumulates in SAR-induced potato plants^13^. Similarly, azelaic acid (AZA) is also derived from C18 fatty acids, and together with its induced protein *AZELAIC ACID INDUCED 1* (AZI1) plays an important role in the systemic immune response^14^. Nevertheless, a recent study presents evidence that methyl salicylate and JA are non-essential for SAR in *Arabidopsis*, and AZA is a general marker for lipid peroxidation rather than a general immune signal^15^,^16^,^17^. Moreover, defective SAR in *acyl carrier protein 4* (*acp4*) mutant plants was also not due to impaired salicylate or JA-mediated plant hormone signalling pathways but was associated with impaired leaf cuticles^18^.

Great efforts have been made to identify other SAR mobile signals, leading to the discovery of a lipid transfer protein, *DIR*1 (ref. 19), which is a *DIR1*-dependent G-3-P derivative^20^. The dehydroabietinal^21^ and the non-protein amino acid pipecolic acid^22^ are translocated signalling molecules. SFD1 and FAD7 function in the synthesis of plastid-synthesized lipids that are critical for SAR^17^,^23^. In addition, certain glycerolipid-dependent factors along with the *DIR1*-encoded lipid transfer protein are essential for long-distance signalling in SAR^17^. Therefore, these data suggest that lipid metabolism is required to establish SAR. However, AZA or JA are oxylipins derived from octadecanoids (C18 fatty acid pathway) and are regarded as dispensable for SAR; thus, other lipids or oxylipins may be involved in SAR signal transmission but have not been discovered. Given the complexity of lipid metabolism, the nature of the mobile signal is still unclear. Although plant oxylipins are important to plant growth and defence, their biological properties are poorly understood. Only one such oxylipin, JA, has been studied to produce a relatively clear account of its anabolism and signal regulation pathway in recent years.

Here we report identification of a gene *SSN*( GhCYP82D) in cotton. Suppressing its expression causes a severe HR-like phenotype in cotton plants. Bioinformatics analysis shows that it belongs to the CYP82 family of cytochrome P450s. The function of SSN was analysed using genetic and metabolic approaches to reveal its role in lesion formation. *SSN* is specifically required for confining the production of a presumed mobile signal involved in systemic cell death by modulating a previously unknown biosynthetic pathway of oxylipins derived from octadecanoids in cotton. We propose that this pathway is involved in SAR signal formation, and these findings suggest a novel metabolic branch that might regulate the JA signalling pathway.

## Results

### Downregulation of GhCYP82D leads to lesion mimic phenotype

**Figure 1:**
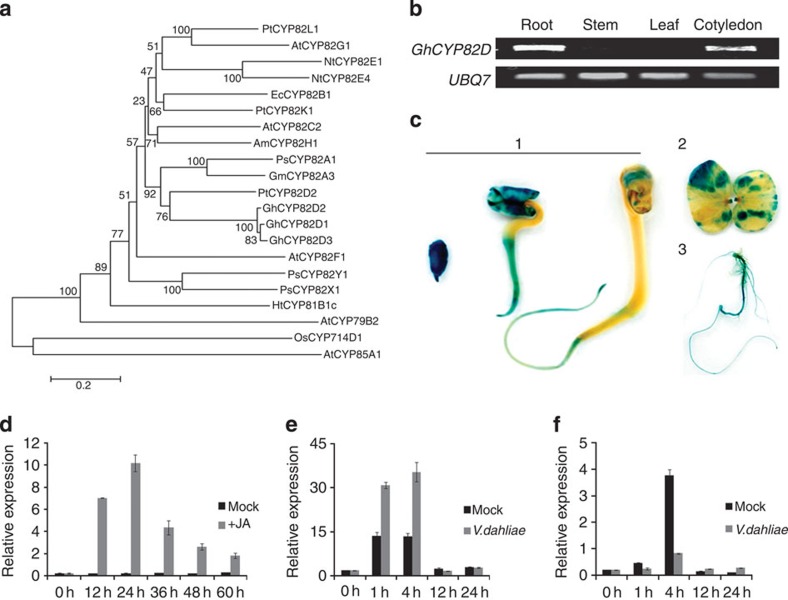
Phylogenetic analysis of the CYP82 family and the *SSN* expression pattern. (**a**) Protein phylogeny of the CYP82 family (P450 enzymes and proteins) from *Gossypium hirsutum* (Gh), *Populus trichocarpa* (Pt), *Pisum sativum* (Ps), *Glycine max* (Gm), *Eschscholzia californica* (Ec), *Ammimajus* (Am), *Arabidopsis thaliana* (At), *Papaver somniferum* (Ps), *Nicotiana tabacum* (Nt), *Helianthus tuberosus* (Ht) and *Oryza sativa* (Os) plants. The neighbour-joining tree was constructed using the MEGA5 program ( http://www.megasoftware.net/). (**b**) RT-PCR analysis of *GhCYP82D* expression in different tissues. Total RNA was isolated from roots, stems, leaves and cotyledons of the WT cotton line YZ1. The *UBQ7* gene was amplified as a control. (**c**) *GhCYP82D1* (*SSN1*) promoter::GUS fusion expression patterns in transgenic cotton. GUS staining was shown in germinating seeds (1), cotyledon (2) and root (3) of young seedlings. (**d**) *GhCY82D* (*SSN*) responses to JA treatments in cv. YZ1. The plants were pretreated with Hoagland’s solution; 25 μM JA was then added to the solution. (**e**) qRT–PCR showing the *V. dahliae*-induced *SSN* expression pattern in the susceptible cotton line Ji11. (**f**) qRT–PCR showing the *V. dahliae*-induced *SSN* expression pattern in the resistant cotton line 7124. The experiments (**d**–**f**) were repeated at least two times with similar results. The values are the means±s.d. for four technical replicates. The transcript levels of each gene were normalized to *UBQ7*.

In our previous work, we isolated an expressed sequence tag from a cDNA library in a screen for genes involved in cotton disease resistance following inoculation with *Verticillium dahliae*^24^. Three highly similar genes (above 94%) were identified in *G. hirsutum* genotype YZ1 each with 1,569-nucleotide open reading frames (ORFs) and putatively encoded proteins of 522 amino acids, with conserved domains that are characteristic of eukaryotic P450 proteins (Supplementary Fig. 1). Sequence analysis revealed that they share 55% identity with PtCYP82D2 but only 48% with AtCYP82C2 (Fig. 1a and Supplementary Fig. 1). Thus, GhCYP82D is a novel P450 subfamily in cotton. The expression profiles were determined using reverse transcriptase–PCR (RT–PCR) with primers for the conserved regions in this gene family. The results showed that they were specifically expressed in roots and cotyledons of seedlings (Fig. 1b), which is consistent with results from glucuronidase (GUS) activity detection using promoters from two family members (Fig. 1c and Supplementary Fig. 2a,b). The gene family is highly induced by multiple phytohormones (Supplementary Fig. 3), including JA (Fig. 1d and Supplementary Fig. 2c), and is induced by *V. dahliae* infection in roots of the susceptible cotton line Ji11 compared with mock treatments (Fig. 1e). However, it is downregulated in the resistant cotton line 7124 (Fig. 1f).

**Figure 2:**
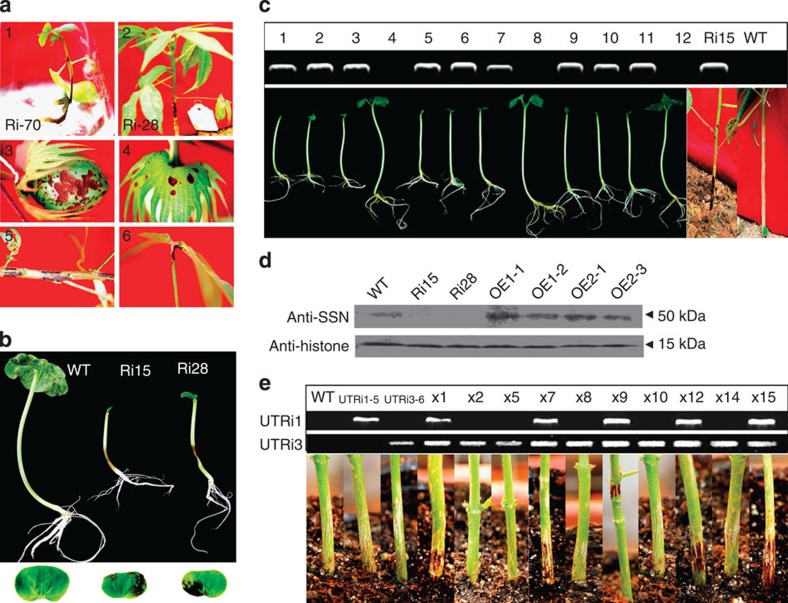
Morphological features of *GhCYP82D* (*SSN*)-RNAi plants grown in soil. (**a**) The lesion phenotype of RNAi transformants, showing *SSN-*RNAi *in vitro* plants (1), T0 plants in field to show lesions on the stem (2), boll shell (3), bract (4), branch (5) and junction with the petiole (6). (**b**) The lesion phenotype on stems and cotyledons of *SSN*-RNAi T1 plants grown for 9 days *in vitro*. (**c**) The cotyledons abscised from approximately 3/4 of the plants cultured for 9 days *in vitro* among the T1 segregations of the *SSN*-RNAi line Ri15. (**d**) Western blotting revealed different expression levels among the transgenic and WT plants leaves. Antibodies were prepared using the same SSN1 and SSN2 protein amino acid sequences as the probes; anti-histone was used as a control. Ri: *SSN*-RNAi plants; OE1: *SSN1*-overexpression plants; OE2: *SSN2*-overexpression plants. (**e**) The F1 plants were derived from a cross between homozygous UTRi3 (*SSN3*)-silenced plant and a heterozygous UTRi1 (*SSN1*)-silenced plant; one gene-specific primer (from 3′-UTR of *SSN1* or 3′-UTR of *SSN3*) and one vector primer (from the 35S promoter) were used to amplify the transgene in F1 plants, plants with both UTRi1 and UTRi3 amplified bands showed a lesion phenotype on the stems.

To explore the role of this P450 subfamily in cotton, we knocked down the expression of the gene family using RNA interference (RNAi) technology for the conserved regions. We also overexpressed the genes of this subfamily using the 35S promoter in transgenic cotton plants (Supplementary Fig. 4). Most *GhCYP82D* RNAi seedlings showed lesion mimics on the stems and most died (Fig. 2a and Supplementary Fig. 5a,b), consistent with the level of gene downregulation (Supplementary Fig. 5c). The gene family was named *SILENCING-INDUCED STEM NECROSIS* (*SSN*), and the three members *GhCYP82D1*, *GhCYP82D2* and *GhCYP82D3* were named *SSN1*, *SSN2* and *SSN3*, respectively. No obvious phenotypic differences were observed between the *SSN1*-, *SSN2*-overexpressing and WT seedlings. A small number of *GhCYP82D* RNAi seedlings (*SSN*-RNAi) survived showing the lesion mimic phenotype on the boll shell, bract and junction between the petiole and leaf in field-grown plants (Fig. 2a and Supplementary Fig. 6a,b). More serious necrosis was observed on the cotyledons when the *SSN*-RNAi-transgenic offspring were aseptically germinated *in vitro*; the cotyledons fell approximately 9 days after germination (Fig. 2b and Supplementary Fig. 7a,b). The lesion mimic reappeared on the stems and cotyledons (Fig. 2b and Supplementary Fig. 7c,d), co-segregating with the downregulation of *SSN* (Fig. 2c). *SSN*-RNAi shoots were rescued by grafting to WT stems to obtain seeds. The transgenic offspring (lines Ri15 and Ri28) showed a strong necrosis phenotype, but the phenotype was inconspicuous for Ri54 (Supplementary Fig. 6c), linked to less *SSN* downregulation (Supplementary Fig. 8a). Low-copy-number insertions of *SSN*-RNAi, *SSN1*-overexpression (OE1) and *SSN2*-overexpression (OE2) plants were examined through western blotting (Fig. 2d and Supplementary Fig. 8b), which confirmed the downregulation and upregulation, respectively.

### The HR-like phenotype is independent of SA synthesis

To further explore the functional redundancy between the three members of the *SSN* family, RNAi-transgenic plants for the three members with specific 3′-UTR regions were generated and characterized by Southern blotting and quantitative RT-PCR (qRT-PCR; Supplementary Figs 4 and 8c–h). Interestingly, the expression compensation was observed in the single-gene-silenced plants between any two members (Supplementary Fig. 8c,e,h). We did not observe any difference in growth between the single-gene-silenced and WT plants. Crossing was employed using different specific 3′-UTR silenced lines to examine the functional redundancy among the gene members. The results showed that silencing any two members of the *SSN* family could induce the lesion phenotype, as verified through an RNAi-segment analysis (Fig. 2e and Supplementary Fig. 9). These results suggest that *SSN* members may have functional redundancy.

**Figure 3:**
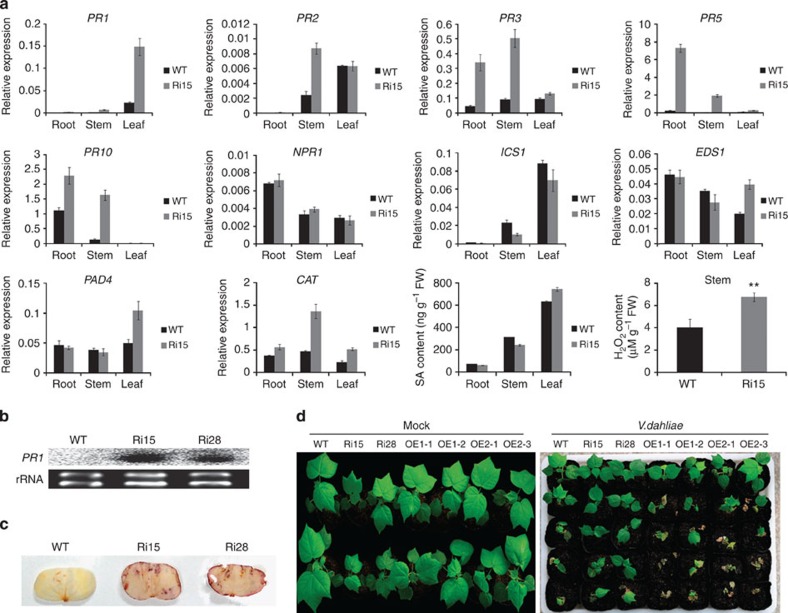
Constitutively activated defence response in *SSN*-RNAi plants and disease resistance symptoms on *V. dahliae* infection. (**a**) The transcript levels of *PR* genes and SA biosynthesis genes and the H_2_O_2_ levels in WT and Ri15 at the 10-day-old seedling before the lesion appeared on the plants. The SA levels of different tissues are from 3-week-old seedlings. The values are the means±s.d. for three biological replicates (**P<0.01, Student’s *t*-test). (**b**) The *PR1* gene expression level was determined using an RNA-gel blot analysis for leaves from 3-week-old seedlings. (**c**) H_2_O_2_ accumulation at the site of lesion formation in *SSN*-RNAi cotyledons was visualized through DAB staining. (**d**) Disease symptoms among the WT, *SSN*-RNAi and OE lines after inoculation with the *V. dahliae* strain ‘V991’. Three-week-old seedlings were root-wounded and dip-inoculated with *V. dahliae* before transplanted to soil. The photos were taken 12 days after inoculation. The experiments were repeated three times with similar results. Shown are representative symptoms of disease (at least 20 plants of each line).

A lesion phenotype is typically associated with cell death during HR to pathogens. HR always triggers the rapid production of reactive oxygen species and induces numerous *PR* genes^25^,^26^ and is typically accompanied by SA accumulation^27^. To evaluate whether the lesion mimic in *SSN*-RNAi plants is due to HR-like cell death, the *PR* genes that are tightly correlated with the HR and H_2_O_2_ level were analysed. The results show that many *PR* genes, including the commonly regarded marker genes *PR1* and *PR5*, were significantly upregulated in the *SSN*-RNAi stem. Further, *CATALASE* (*CAT*) was upregulated, and excess levels of H_2_O_2_ were detected in the *SSN*-RNAi stem (Fig. 3a), which suggests that silencing *SSN* constitutively activates the HR in cotton seedlings. However, *NPR1* expression was not influenced by *SSN* silencing in cotton seedlings. Moreover, the genes involved in SA biosynthesis, such as *ICS1*, *EDS1* and *PAD4*, showed similar expression pattern to *NPR1*, and we did not observe differences in SA levels between WT and *SSN*-RNAi seedling stems (Fig. 3a). In addition, *PR1* upregulation was detected through RNA-gel blotting in different silenced lines (Fig. 3b), and excessive levels of H_2_O_2_ shown by 3, 3′-diaminobenzidine (DAB) and 2′,7′-DCFDA staining were detected in the *SSN-RNAi* cotyledons and stems, further followed by necrosis and collapse of vascular bundle cells (Fig. 3c, Supplementary Fig. 10). To investigate the involvement of *SSN* in the cotton response to *V. dahliae*, seedlings were inoculated with the *V. dahliae* strain ‘V991’. Compared with WT, resistance to *V. dahliae* was improved in *SSN*-RNAi but attenuated in OE seedlings (Fig. 3d).

These data suggest that *SSN*-RNAi seedlings exhibit a constitutively activated HR-like cell death phenotype, but this is independent of SA synthesis.

### SSN confers disease resistance by regulating JA anabolism

**Figure 4:**
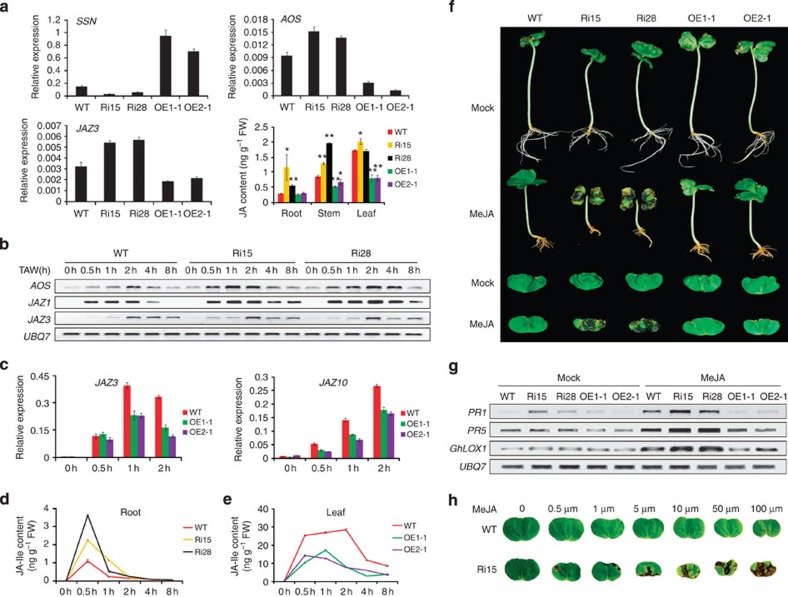
SSN regulates the JA synthesis and signal transduction pathways. (**a**) Selected JA synthesis and signal transduction pathway genes from the RNA-Seq data were verified through qRT-PCR. RNA was extracted from roots of 6-day-old seedlings. Error bars indicate s.d. from four technical replicates. The JA levels were constitutively enhanced in 3-week-old *SSN*-RNAi seedlings. The values are the means±s.d. for three biological replicates. The asterisks indicate statistically significant differences between the transgenic and WT plants (*P<0.05, **P<0.01, Student’s *t*-test). (**b**) Time course for *AOS* and *JAZ* mRNA accumulation analysed using RT–PCR in wounded roots of WT and *SSN*-RNAi. The total RNA was isolated from wounded roots of 3-week-old WT and *SSN*-RNAi plants. The cotton *UBQ7* gene was amplified as a control. TAW, time after wounding. (**c**) qRT–PCR amplification of *JAZ* genes after wound treatment. Total RNA was extracted from 4-week-old WT and OE plants. Error bars indicate s.d. from four technical replicates. (**d**,**e**) Kinetics of wound-induced JA-Ile levels in roots and leaves. The values are the means±s.d. for three biological replicates. (**f**) Morphological phenotype shows that WT and transgenic seedlings grown for 5 days on media containing MeJA (5 μM) are sensitive to JA-induced growth inhibition. *SSN*-RNAi plants show more sensitivity to JA-mediated growth inhibition and exhibited accelerated and aggravated HR-like necrosis. (**g**) RT–PCR analysis of marker genes associated with HR in transgenic seedling cotyledons grown for 2 days on MeJA (5 μM) media. The cotton *UBQ7* gene was amplified as a control. (**h**) Photographs of WT and the Ri15 line grown for 4 days on media containing different MeJA concentrations.

To determine the function of *SSN* in the cotton immunity system, Illumina sequencing was employed to identify genes that were differentially expressed in roots between the *SSN*-RNAi (Ri15) and WT seedlings. We isolated 184 genes with different expression patterns between the two lines (RPKM>15 and the absolute value of log2 ratio >1 based on the false discovery rate (FDR) <0.001, Supplementary Table 1). Of these, 32 genes did not have corresponding homologous genes in *Arabidopsis*, and annotations for approximately 40 homologues in *Arabidopsis* are unknown. Approximately one-sixth of the remaining 112 genes have been shown to directly participate in JA and SA signalling pathways. Interestingly, all the genes involved in JA biosynthesis, such as *LOX2*, *AOS* and *AOC4*, and the signalling pathway, such as *MYC2-like*, *JAZ1*, *JAZ3* and *JAZ10*, were upregulated in *SSN*-RNAi. However, the WRKY transcription factors involved in the SA signalling pathway, such as *WRKY40*, *WRKY50*, *WRKY51* and *WRKY70*, were downregulated in *SSN*-RNAi seedlings (Table 1 and Supplementary Table 1). Certain genes were verified through qRT-PCR, and the results were consistent with the Illumina sequencing (Supplementary Fig. 11a). Silencing *SSN* in cotton upregulated genes related to JA biosynthesis, while the overexpression of *SSN* downregulated the expression level of genes involved in JA biosynthesis and decreased JA levels in leaves and stems (Fig. 4a).

JA plays a role in the plant wound response. The expression patterns for genes involved in the JA signal pathway were examined in seedlings after experimental wounding. The results show that *AOS*, *JAZ1* and *JAZ3* expression levels were more rapidly induced in *SSN*-RNAi than WT seedlings (Fig. 4b). However, *JAZ3* and *JAZ10* transcripts were apparently attenuated in OE lines after a wound treatment compared with WT seedlings (Fig. 4c). In addition, we measured the jasmonoyl-L-isoleucine (JA-Ile) levels, which is the activated form of JA. Consistent with the kinetics of gene expression, *SSN*-RNAi plants hyperaccumulated JA-Ile levels compared with WT plants at the 0- to 2-h time points after wounding (Fig. 4d). In contrast to the elevated JA-Ile levels in silenced plants, lower JA-Ile levels in the OE lines correlated with lower gene expression (Fig. 4e). This shows that JA biosynthesis was partially blocked by the *SSN* overexpression, and wound signal transduction was also weakened due to the reduced JA.

To further determine whether SSN are P450 enzymes that are directly involved in the catalytic effect on JA, 5-μM MeJA was applied into the MS medium. The seedling root growth for the WT and OE seedlings did not differ. However, *SSN*-RNAi seedlings showed an increased response to the exogenous MeJA treatment, with clear root growth inhibition and hypocotyl elongation retardation associated with constitutively active JA synthesis (Fig. 4f). Similar results were obtained using 1-μM MeJA (Supplementary Fig. 11b). Moreover, exogenous MeJA treatment accelerated and aggravated HR-like necrosis on *SSN*-RNAi cotyledons (Fig. 4f). Further, the expression levels of marker genes associated with HR were upregulated more intensively for *SSN*-RNAi during the MeJA treatment compared with the WT. In contrast, the expression levels for these genes were attenuated in the OE seedlings (Fig. 4g). In addition, with the increase in MeJA concentration, *SSN*-RNAi cotyledon necrosis was more dramatic and spread more rapidly, but necrosis was not observed in WT (Fig. 4h). AZA, which is the metabolite from the oxylipin pathway and was identified as a mobile signal of SAR^14^, upregulated the expression of *SSN* and suppressed the expression of *AZI1* which was required for SAR in the roots of cotton seedlings (Supplementary Fig. 12a,b). HR-like necrosis in *SSN*-RNAi was accelerated and aggravated by an exogenous AZA treatment (Supplementary Fig. 12c). With increasing AZA concentrations, necrosis was observed in the cotyledon and the *SSN*-RNAi seedlings died rapidly (Supplementary Fig. 12d).

In addition to the 13-LOX gene (*LOX2*), which is involved in JA biosynthesis^28^, two other 9-LOX genes (*LOX1* and *LOX5*; Supplementary Fig. 11a) in the JA biosynthesis branch^29^ were found in the RNA-seq data, but the precise metabolic pathways are unclear^30^. However, previous result showed that *GhLOX1* was involved in the cotton HR^31^.

### SSN is involved in fatty acid and oxylipin metabolism

**Figure 5:**
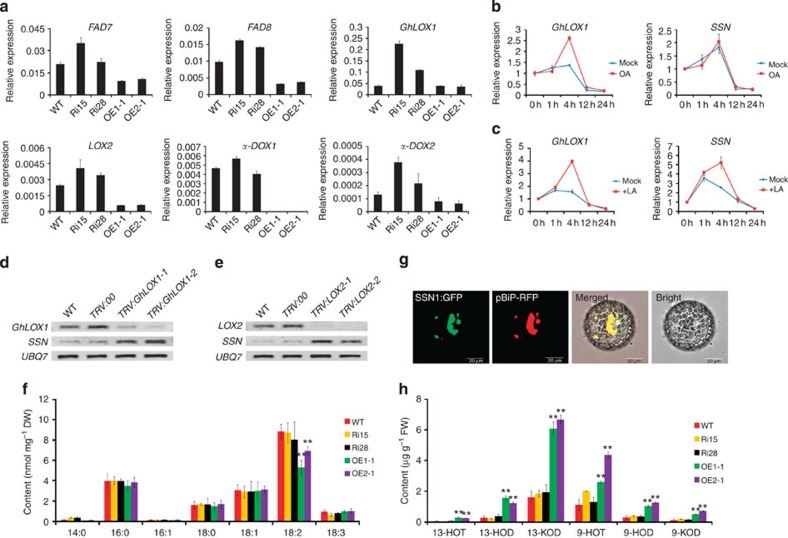
SSN significantly modulates the metabolism of oxylipins derived from the octadecanoid pathway. (**a**) The key genes selected from 18:2 fatty acid metabolism pathway showed different expression patterns in the *SSN*-RNAi and OE lines. RNA was extracted from roots of 6-day-old seedlings. Error bars indicate s.d. from four technical replicates. The transcript levels of each gene were normalized to *UBQ7*. (**b**,**c**) The *GhLOX1* and *SSN* expression patterns responded to the oleic acid (OA) and linoleic acid (LA) treatments in cotton cotyledon, as demonstrated using qRT–PCR. RNA was extracted from seedlings at different intervals after an OA (10 mM) or LA (10 mM) application. Error bars indicate s.d. from four technical replicates. (**d**) RT–PCR analysis of *SSN* expression levels in WT, *TRV:00* and *TRV:GhLOX1* leaves. (**e**) RT-PCR analysis of the expression levels of *SSN* in WT, *TRV:00* and *TRV:LOX2* leaves. (**f**) Fatty acid composition in the WT and transgenic line cotyledons. The values are the means±s.d. for four biological replicates. The asterisks indicate statistically significant differences between the transgenic and WT plants (**P<0.01, Student’s *t*-test). (**g**) Subcellular localization of the *SSN1* gene product. From left to right: the protoplast showed green fluorescent signal at 488 nm; the same protoplast showed red fluorescent signal at 561 nm; image of the green and red signals; bright-field image. The data represent transformed protoplasts. The green and red fluorescent signals were examined 24 h after transformation. At least three independent transformation experiments were performed using the two constructs. Scale bars, 20 μm. (**h**) Major oxylipin compositions for 9/13 hydroxy-FAs and keto-FAs in the WT and transgenic line leaves. 9- or 13-HOT: 9- or 13-hydroxy octadecatrienoic acid; 9- or 13-HOD; 9- or 13-hydroxy octadecadienoic acid; 9- or 13-KOD; 9- or 13-keto octadecadienoic acid. The values are the means±s.d. for three biological replicates. The asterisks indicate statistically significant differences between the transgenic and WT plants (*P<0.05, **P<0.01, Students *t*-test).

Previous studies have shown that constitutively activated JA biosynthesis and signalling do not induce lesion mimics^32^; however, certain oxylipins mediate reactive oxygen species production and cell death in plants^33^,^34^. The results above suggest that SSN may be involved in oxylipin metabolism. We characterized the expression profiles for genes involved in the octadecanoid pathway in transgenic plants (Fig. 5a and Supplementary Fig. 13a). The results show that the expression levels of genes involved in 18:2 fatty acid metabolism, such as *FAD7*, *FAD8*, *GhLOX1*, *LOX2*, *a-DOX1* and *a-DOX2* were significantly inhibited in OE seedling roots compared with WT and *SSN*-RNAi (Fig. 5a). We applied individually oleic acid (OA, 18:1), linoleic acid (LA, 18:2) and α-linolenic acid (ALA, 18:3) to WT seedlings, and examined the *SSN* expression pattern. *SSN* expression patterns were the same as *GhLOX1*, which uses LA and ALA as substrates (Fig. 5c and Supplementary Fig. 13b). Nevertheless, OA could induce *GhLOX1* expression, but not *SSN* expression (Fig. 5b). Further, *SSN* was upregulated in cotton seedling roots when *GhLOX1* and *LOX2* were silenced, individually, through virus-induced gene silencing (Fig. 5d,e).

To assess the metabolic differences between the *SSN*-RNAi and OE lines, fatty acid levels were determined. Interestingly, only LA levels were significantly reduced in the OE seedlings compared with WT and *SSN*-RNAi (Fig. 5f). Furthermore, SSN-green fluorescent protein fusion protein localization indicates that SSN1 is an endoplasmic reticulum-localized protein (Fig. 5g). Further, transmission electron microscopy analyses on WT and transgenic seedling cotyledons suggest that *SSN* expression may influence plasmid development (Supplementary Fig. 13c). The cotyledon oxylipins were also measured, and 9-HOD/T, 13-HOD/T and 9/13-KOD dramatically accumulated in the OE seedlings (Fig. 5h). These data indicate that SSN is involved in the octadecanoid pathway.

### HR-like phenotype is related to imbalance in LOX pathway

**Figure 6:**
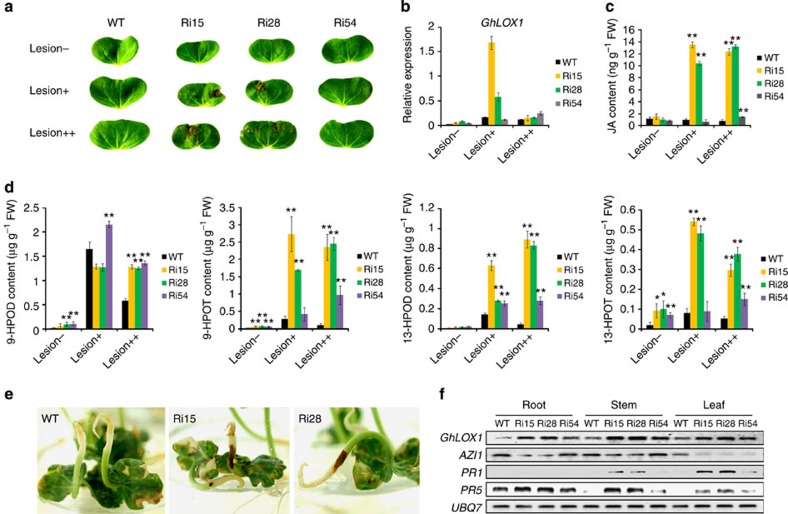
The 9-LOX pathway plays an important role in systemic cell death. (**a**) Shown are different stages of lesion formation: 5-day-old seedlings before the lesion appears (lesion−), 7-day-old seedlings when the lesion appears (lesion+) and 8-day-old seedlings after the lesion forms (lesion++). (**b**) Transcript levels of the 9-LOX gene *GhLOX1* at the different lesion formation stages shown in **a**. Total RNA was extracted from cotyledon as shown in **a** and subjected to a qRT-PCR analysis. (**c**) JA levels stood for 13-LOX (*LOX2*) pathway activation in cotyledon tissue from the same set of plants used in **a**. (**d**) The products (9- or 13-HPOD/T) of the 9-LOX or 13-LOX enzymes in the cotyledon tissue from **a**. The values (**b**–**d**) are the means±s.d. of three biological replicates. The asterisks indicate statistically significant differences between the transgenic and WT plants (*P<0.05, **P<0.01, Student’s *t*-test). (**e**) Germinating *SSN*-RNAi seeds on the medium where the roots were not placed into medium manually also show a lesion phenotype on the stems. (**f**) The *GhLOX1*, *AZI1*, *PR1* and *PR5* gene transcript levels in different tissues (root, stem and leaf) of *SSN*-RNAi plants before the lesion phenotype appears. Total RNA extracted from different tissues of 3-week-old seedlings was subjected to RT–PCR analysis.

LOX-dependent hydroperoxide fatty acid formation is necessary for hypersensitive cell death development in plants, such as cotton^31^,^35^, pepper^36^ and tobacco^37^. Because OE seedlings accumulate more hydroxy and keto C18 fatty acids, SSN likely acts on C18 fatty acids and competes for substrates with LOXs. We therefore hypothesized that SSN might modulate fatty acids metabolism in a novel pathway that differs from the pathway LOXs are involved in, and SSN inhibition leads to free fatty acid accumulation, which enhances *LOXs* expression and results in lipid peroxidation. To investigate this hypothesis, cotyledons from seedlings after germination at different lesion mimic formation stages were harvested for gene expression and hydroperoxide fatty acid composition analyses (Fig. 6a). The results show that *SSN* silencing strengthens the other two oxylipin metabolism pathways by forming 9-HPOs from 9-LOX and 13-HPOs from 13-LOX. During cotyledon development, *GhLOX1* expression was constitutively active and reached a maximum as the lesion was observable in cotyledons (Fig. 6b). Moreover, the levels of oxylipins, 9-HPOD/9-HPOT metabolized by GhLOX1 (9-LOX) and 13-HPOD/13-HPOT metabolized by LOX2 (13-LOX) accumulated dramatically during *SSN*-RNAi cotyledon development (Fig. 6d). Consequently, the genes involved in the JA biosynthesis pathway were constitutively activated, and the JA levels clearly increased in *SSN*-RNAi cotyledons (Fig. 6c and Supplementary Fig. 14).

It is difficult to understand why the lesion could form on the stems, where the *SSN* gene expression was not detected, but the expression was rich in roots, where lesions were not observed. To investigate this, seeds were germinated *in vitro* without placing the roots into the medium manually, to allow the roots access to oxygen, but lesions were still not observed on the roots (Fig. 6e). We assume that a lipid-derived substance or signal in the roots may exist, and this signal could be transported to, and excessively accumulate in, the stems and this leads to lesion formation.

To test this hypothesis, root, stem and leaf tissues were harvested from silenced and WT plants for biochemical analyses. The results show that *GhLOX1* and *PR5* transcripts were significantly upregulated in the stems and leaves compared with the *SSN*-RNAi seedling roots. In addition, high *PR1* expression levels were only detected in *SSN*-RNAi leaves, and the *AZI1* expression level was downregulated before lesion formation (Fig. 6f). We then performed a grafting study using WT and *SSN*-RNAi. SAR marker genes, such as *PR1* and *PR5*, were dramatically upregulated in leaves when the WT shoots were grafted onto stocks with *SSN*-RNAi as the rootstocks compared with the control, WT shoots grafted onto WT rootstocks. Further, the *GhLOX1* expression pattern showed a similar trend to the *PR* genes, and the *AZI1* expression level was downregulated in the heterografted WT::*SSN*-RNAi leaves. However, we did not observe a significant difference in the leaf *NPR1* and *SSN* expression levels after grafting (Supplementary Fig. 15).

## Discussion

Cytochrome P450s are one of the largest plant protein families, and are involved in a wide range of primary and secondary metabolic biosynthesis pathways. According to phylogenetic studies, P450s in the plant kingdom are divided into 10 separate clans in 61 families^38^. In this study, we isolated and characterized a novel P450 subfamily, CYP82D (SSN), in cotton. However, little information is available about the biochemical functions of the CYP82D subfamily in plants. Members of the CYP82 family (Fig. 1a) respond to stress in pea, tobacco and soybean^39^,^40^,^41^. Recent studies show that the *CYP82* gene plays a role in *Arabidopsis* pathogen and herbivore resistance^42^,^43^, which led us to speculate that SSN may be involved in a specific area of secondary metabolism that is important for abiotic and biotic stress. Here we show that *SSN*-RNAi plants display spontaneous HR-like cell death with high *PR* gene expression. However, content analysis shows that the cell death phenotype is independent of a high level of endogenous SA, which is typically thought of as an essential condition for lesion mimic formation in many *Arabidopsis* mutants. We therefore speculate that cotton HR is regulated by a mechanism, which is independent of the SA pathway. *SSN* is highly expressed in cotyledons, and *SSN* silencing led to excessive JA accumulation, which implies that *SSN* may participate in lipid metabolism. Identifying *SSN* provides new insight into the function of a P450 subfamily, for which the physiological role in plants hitherto remains largely unknown. In addition, these data are helpful for discerning the relationship between the HR and JA or lipid metabolism, especially through the octadecanoid pathway.

Generally, the cell death with lesion mimic phenotype was thought as a typical symptom of the HR in plant disease resistance. It is a suicide mechanism response to pathogen infection, similar to PCD in animals^44^. Previous studies show that animal and plant cells share a similar signal transduction pathway that triggers apoptosis and is derived from lipoxygenase activation^45^. Animal oxylipins have been associated with the inflammatory response and apoptotic pathway^46^. Therefore, elucidating the cellular processes that govern oxylipin homeostasis derived from lipoxygenase is essential for understanding the plant HR- and defence-related processes. Here we show that SSN regulates HR in cotton by modulating oxylipin metabolism. Metabolite analysis indicates that SSN defines a major pathway for lipid catabolism in the seedling stage. Given the JA-induced and wound-induced *SSN* expression, SSN activity may be involved in the JA pathway. The observed hydroperoxy fatty acid overproduction in *SSN*-RNAi cotyledons indicates that this pathway governs a negative regulatory mechanism, which effectively restrains uncontrollable hydroperoxide accumulation. This possibility is consistent with the time course for wound-induced JA-Ile derived from 13-hydroperoxy fatty acids, which shows greater accumulation in silenced plants but lower accumulation in OE plants on tissue damage. JA biosynthesis was partially blocked by SSN overexpression. Further, SSN co-expresses with the gene *GhLOX1*, which is involved in LA and ALA metabolism as well as induced by LA and ALA treatment. Silencing *SSN* could induce *LOX* expression, and meanwhile, SSN expression was induced when the LOXs are silenced. As expected, significantly reduced levels of the fatty acid 18:2 were detected in overexpressing plants followed by over-accumulation of hydroxy and keto fatty acids. Our results support a hypothesis that SSN could act in a certain way for the metabolism of fatty acid-derived secondary metabolites by modulating JAs and fatty acid hydroxides synthesis.

Grafting experiments are typically used to discern whether substances or signals are transferred from the root to shoots. Grafting the WT shoots to the *SSN*-RNAi rootstocks showed that certain substances or signals may be generated by the *SSN*-RNAi roots and transmitted to the WT shoots, which leads to an activated defence response. Moreover, when the seedlings were grown freely *in vitro* with oxygenated roots, the lesions only appeared on the stems, not the roots. This implies that there may be a mobile signal produced in the roots that triggers systemic cell death in the stems. SSN may be required to regulate the mobile signal generation and accumulation; seemingly, this signal is derived from the LOX pathway and positively regulates LOX expression.

LOX-dependent hydroperoxide fatty acid formation is necessary for hypersensitive cell death development in tobacco^33^,^47^. GhLOX1 mediates cell death during the cotton HR when it interacts with Xcm (*Xanthomonas Campestris* pv. *Malvacearum*)^31^,^35^. The pepper 9-lipoxygenase gene *CaLOX1* positively regulates defence responses and hypersensitive cell death^36^. Thus, GhLOX1 plays a central role as positive regulator of HR. Further, fatty acid hydroperoxides, LOX products, can induce PCD in tomato^48^ and a recent study showed that oxylipin metabolism is a critical pathway that positively regulates PCD process during compatible interactions^37^. However, no oxylipins have been reported to induce HR as transferable elements or signal molecules.

Our study suggests HPOD/HPOT may not only act as a substrate for either HPL or AOS to drive the GLV or JA pathway, but they or another unknown compound derived from them may also act as a long-distance mobile signal for systemic cell death. Moreover, they induced HR and *PR* gene expression. However, it is difficult to understand how the substance or signal was transferred. Recent work has shown that JA and AZA, which belong to this oxylipin pathway, may operate as the mobile signals^12^,^14^; however, several studies disagree with those results^15^,^16^. Although the precise compounds have not been identified, more signal molecules have been documented, such as the lipid transfer protein *DIR1*, a G3P-dependent signal, glycerolipid, cuticle and 12-oxo-phytodienoic acid^13^,^17^,^18^,^19^,^20^. Few studies have provided links between these putative signals. Our studies raise the hypothesis that an oxylipin-derived factor of C18 fatty acids is not only required for systemic cell death but also may partially affect the transmission of SAR signalling. Thus, JA or AZA may result from the SAR generation process, not form the basis for SAR signalling transmission.

Furthermore, glycerol may promote lesion mimic formation and *PR* gene expression on silenced plant cotyledons (Supplementary Fig. 16). Previous studies have shown that oxylipins occur not only in free form but can also be bound to phospholipids, glycolipids and neutral lipids^49^. On the basis of our results, we propose that suppressed *SSN* expression leads to an upset in the balance of oxylipin homeostasis, which in turn controls the ‘on/off’ signal for SAR.

Traditionally, plants were thought to protect themselves against biotrophic and necrotrophic pathogens through distinct mechanisms, with the SA pathway generally effective against biotrophic or hemibiotrophic pathogens. The *SSN*-RNAi plants show resistance to *V. dahliae*, which is a hemibiotrophic pathogen that causes serious vascular disease in cotton on infection in underground root tissue^50^. However, the SA signalling pathway was suppressed by constitutively activated JA pathway in *SSN*-RNAi roots. This observation is explicable based on our studies.

First, *SSN* was downregulated in the resistant cotton line but upregulated in the susceptible cotton line on *V. dahliae* infection. Although the specific mechanism of disease resistance has been unclear until now, *SSN* silencing in the susceptible cotton line YZ1 is the same as in resistance response of the downregulation process in resistant cotton line 7124. Second, we inoculated cotton seedlings by dipping the wound roots into a *V. dahliae* spore suspension, and greater JA-Ile accumulation was documented in wounded roots of *SSN*-RNAi plants (Fig. 4d). Further, recent research has shown that the JA pathway is important to *V. dahliae* infection^51^,^52^. Thus, we propose that JA, rather than SA, plays a key role in cotton *V. dahliae* resistance.

## Methods

### Plant materials and growth conditions

The cotton plants *Gossypium barbadense* cv. 7124, *Gossypium hirsutum* cv. Ji11 and *Gossypium hirsutum* cv. YZ1, as well as transgenic lines derived from YZ1 in these experiments, were cultivated in Wuhan, China under normal farming practices or grown in the greenhouse during the winter. The greenhouses were maintained at a temperature of 28–35/20–28 °C day/night. The roots, stems, cotyledons and leaves were collected from seedlings cultured in a growth chamber or in Hoagland’s solution. The collected materials were preserved at −80 °C until required for further analysis.

### Plant treatments

For the JA/MeJA or AZA treatments using seedlings grown in Hoagland’s solution, 25 μM JA, 50 μM MeJA or 1 mM AZA (Sigma) was added to the Hoagland’s solution for the WT YZ1 plants (3 weeks), and the controls were added with ethanol to 0.01 or 0.2%. For the SA or ACC treatment, the cotton line YZ1 (3 weeks old) was grown in Hoagland’s solution, and 0.5 mM SA or 5 μM ACC (Sigma) was added; water was added to the controls. For the MeJA treatment in the medium, the WT and transgenic cotton lines were grown on a half-strength MS medium for 1.5 days and were then transferred to a mock treatment (0.01% ethanol) and media containing different concentrations of JA. For the AZA treatment on the medium, the WT and transgenic cotton lines were grown on a half-strength MS medium for 1.5 days; they were then transferred to a mock treatment (0.1% ethanol) and media containing the required AZA concentrations. For the glycerol treatment on the medium, the WT and transgenic cotton lines were grown on half-strength MS medium for 1.5 days; they were then transferred to a half-strength MS medium supplemented with or without 0.5% glycerol. For the FA treatment, FA stock solutions (200 mM) were prepared in 80% ethanol. Working solutions (10 mM) were prepared in deionized water with the surfactant SilwetL77 (0.02%). Mock solutions were prepared with ethanol (1%) and surfactant (0.02%) in deionized water. Cotyledons from the WT cotton line YZ1 (1 week old) were grown in a conical flask and sprayed with mock treatment or 10 mM various FAs (OA, LA and ALA), respectively. The tissues were then harvested at the indicated time intervals for RNA extraction.

### Wound treatment

For silenced plants, the taproots of 3-week-old plants were grown in Hoagland’s solution and cut to lengths of ~1 cm using scissors. At various time points after wounding, the roots (four or five plants) were harvested, immediately frozen in liquid nitrogen and stored at −80 °C until they were used for RNA or JA extraction. For the overexpressing plants, fully expanded true leaves of 4-week-old plants were wounded to ~40% of the leaf area by crushing the leaf across the midrib using a haemostat. The leaves (four or five plants) were harvested as described above.

### Pathogen infection

*G. barbadense* cv. 7124 and *G. hirsutum* cv. Ji11 seeds were grown in commercial sterilized soil for 3 weeks. The plants were inoculated with the *V. dahliae* strain V991, as described previously^24^. The plant roots were harvested for RNA extraction at 0, 1, 4, 12 and 24 h after inoculation. The control plants were treated with water and sampled at the same time points. Roots from five individual seedlings were collected for each treatment at each sampling time point. Roots from 3-week-old Hoagland’s solution-cultured WT and transgenic plant seedlings were separately wounded with scissors and dip-inoculated with the *V. dahliae* strain V991 to observe the disease symptoms.

### *SSN* cloning and sequence analysis

Total RNA was extracted from cv. YZ1 or cv. 7124 roots using the guanidine thiocyanate method^53^. The first strand of cDNA was synthesized using the SuperScript III reverse transcriptase (Invitrogen, Carlsbad, CA, USA). The expressed sequence tag sequence was isolated from a suppression subtractive hybridization library of cotton line 7124 (ref. 24). The full-length sequence was obtained through 5′- and 3′-rapid amplification of the cDNA end (5′- and 3′-RACE) in accordance with the GeneRacer Kit user manual (Invitrogen) using cv. YZ1 cDNA as the PCR template. The ORF was predicted using ORF Finder ( http://www.ncbi.nlm.nih.gov/gorf/gorf.html). Three genes with highly similar sequences were found during the sequencing process, which are referred to as *GhCYP82D1*, *GhCYP82D2* and *GhCYP82D3*. The primer sequences are listed in Supplementary Table 2. A sequence similarity analysis was performed using BOXSHADE software ( http://www.ch.embnet.org/software/BOX_form.html), and the sequence alignments were completed using the ClustalX and MEGA5 software with the neighbour-joining method. The *GhCYP82D1* and *GhCYP82D2* promoter sequences were obtained through DNA walking in accordance with the Genome Walker Universal Kit User Manual (Clontech) using cv. YZ1 DNA as the PCR template.

### Plasmid construction and plant transformation

The conserved region of the three *SSN* genes and 3′-UTR-specific regions of each gene were individually selected as the RNAi target and cloned into the RNAi vector pHellsgate 4 using the gateway system. Full-length *SSN1* and *SSN2* genes were amplified from the cv. YZ1 cDNA and cloned into pK2GW7,0 (Ghent University). The *SSN1* and *SSN2* promoters were fused with the GUS reporter gene in pGWB433 (Research Institute of Molecular Genetics, Shimane University, Matsue, Japan). The expression constructs were introduced into cv. YZ1 through the *Agrobacterium tumefaciens* strains LBA4404 or EHA105 (ref. 54). The TRV vectors were constructed, and the *Agrobacterium tumefaciens* (GV3101) were prepared for virus-induced gene silencing in accordance with a previous study^51^. The sequences used to construct *TRV:GhLOX1* and *TRV:GhLOX2* were amplified from the cv. YZ1 cDNA. The PCR fragments were digested with BamHI and KpnI and then ligated into the *TRV:00* plasmid. The primer sequences are listed in Supplementary Table 2. The vectors were used to transform *A. tumefaciens* through electroporation. The *A. tumefaciens* with TRV vectors were infiltrated into the 10-day-old YZ1 seedling cotyledons. The seedlings were then grown at 25 °C with a 16/8 h light/dark photoperiod in an incubator.

### Illumina sequencing analysis

Total RNA was extracted using a plant total RNA kit (Sigma, St Louis, MO, USA) from 6-day-old Ri15 line and WT control seedling roots grown under identical conditions on a half-strength MS medium. RNA sequencing and data analysis were performed by the Beijing Genomics Institute (BGI, Shenzhen, China) using the Illumina Genome Analyzer. Briefly, the cDNA was digested with NlaIII and then ligated with the first adaptor containing the recognition site of MmeI, a type II endonuclease that cleaves at sites 21 bp from the recognition site. After digestion by MmeI, the transcripts were ligated with the second adaptor. Single-end sequencing mode was used and the read length was 49 bp. A sequence data set was constructed from cotton unigenes using NCBI ( http://www.ncbi.nlm.nih.gov/unigene/?term=txid3633[Organism:exp]), which was used as the reference database. SOAPaligner/soap2 method was used to map reads and then the expression level of each gene was normalized to RPKM (reads per kb per million reads. The differential gene expression between WT and Ri15 was determined by taking the log2 ratio of RPKM. The Audic and Claverie method was used for normalization procedures and statistical data analysis^55^. FDR was used to determine the threshold of *P* value in multiple test and analysis. The RPKM>15 in any one sample and the absolute value of log2 ratio >1 based on the FDR<0.001 were used as the threshold to judge the significance of gene expression difference.

### Histochemical assay

For GUS detection, fresh tissues were collected from the *SSN1* promoter::GUS plants and *SSN2* promoter::GUS plants, dipped into pre-chilled 80% (v/v) acetone for 30 min, infiltrated into a staining solution under a vacuum for 15 min and then moved to 37 °C for 6 h. The staining solution was composed of 0.9 g l^−1^ 5-bromo-4-chloro-3-indolyl-b-glucuronic acid, 50 mM sodium phosphate buffer (pH 7.0), 20% (v/v) methanol and 100 mg l^−1^ chloromycetin. The samples were successively washed with 75% ethanol and then examined and photographed with a Nikon D40 camera (Japan). For H_2_O_2_ detection, the stems without a lesion phenotype in *SSN*-RNAi and WT plants were sampled for H_2_O_2_ measurements as described previously^56^. For DAB staining, *SSN*-RNAi plant cotyledons were incubated in 1 mg ml^−1^ pH 3.8 DAB-HCl (Sigma-Aldrich, USA) in the dark for 8 h. The cotyledons were then cleared by boiling in alcoholic lactophenol (95% ethanol:lactophenol, 2:1 v/v) for 20 min. The reddish colour of the cotyledons was used as evidence of H_2_O_2_ and visualized using a Nikon D40 camera (Japan). For 2′,7′-DCFDA staining, the hypocotyls of each lines were excised from 9-day-old seedlings, which have no lesion phenotype on stems on media and cut into about 5-mm segments, then incubated for 30 min in the dark at 30 °C in 2′,7′-DCFDA diluted with phosphate buffer solution to 10 μM. The images were observed and recorded using an Olympus light microscope equipped with an Olympus U-PMTVC adapter and a Leica DC300F camera. For section observation, the hypocotyls of each lines were excised from 9-day-old seedlings, which have the weak lesion spot on stems on media and cut into 5–7 mm segments, and then were fixed in FAA solution (formaldehyde:acetic acid: 70% alcohol:water, 1:1:10:8) overnight at 4 °C. Samples were dehydrated in a progressive series of ethanol dilution, infiltrated in chloroform overnight at 37 °C and embedded in paraffin wax (melting point 56 °C). Sections (8 μm) were prepared using a rotary microtome (KD 2058, KEDEE, China) and stained with Safranin fast green. The sections were observed and photographed under a photomicroscope (DM2500, Leica, Wetzlar, Germany). More than 10 samples of each line were analysed above.

### Transmission electron microscopy scanning

WT and transgenic seedling cotyledons were grown for 6 days under identical conditions, cut into 1 mm^2^ pieces with a scalpel, prefixed in the fixing solution (2.5% glutaraldehyde adjusted to pH 7.4 with 0.1 M phosphate buffer and fixed in 2% OsO4 in the same buffer) and then cut into smaller pieces with scissors. Ultrathin sections were obtained using a Leica UC6 ultramicrotome (Leica, Germany) and stained with uranyl acetate then with lead citrate. The images were observed and recorded using a HITACHI H-7650 transmission electron microscope (Hitachi High-tech, Ibaraki-ken, Honshu, Japan) at 80 KV and a Gatan 832 CCD camera (Gatan, Pleasanton, CA).

### Quantitative real-time reverse transcription-PCR

The total RNA was isolated as previously described^57^. Tissues were ground in a mortar with liquid nitrogen and ice-cold extraction buffer containing 1% β-mercaptoethanol was added and mixed completely by inverting the tube. The supernatant was purified by phenol and chloroform. The RNA was precipitated by isopropanol and sodium acetate (3 M) and then washed by 75% ethanol. Air-dried RNA was dissolved in diethylpyrocarbonate-treated water. The RNA was reverse transcribed to cDNA using the SuperScript III reverse transcriptase (Invitrogen). Quantitative real-time (qRT) PCR was performed using the ABI Prism 7000 system (Applied Biosystems, Foster City, CA, USA). For qRT-PCR analysis, at least 5–10 plants of every line or treatment were sampled for each independent biological replicate. Gene sequences were obtained from the public NCBI UniGene data bank. The primers are listed in Supplementary Table 2. Four technical replicates or three biological replicates for each experiment were performed. Error bars represent the s.d. *UBQ7* was amplified as a control. The expression value of all genes was normalized by referring to *UBQ7* as ‘1’.

### Southern, northern and western blotting

Genomic DNA was extracted from young leaves of transgenic cotton lines using the plant genomic DNA kit DP305 (Tiangen Biotech, Beijing). The *NPTII* gene was used as a probe for Southern blotting to detect transgene insertion. Total RNA from the leaves of 3-week-old seedlings was extracted as described above; a *PR1* fragment was used as the probe for northern blot analysis in *SSN*-RNAi plants. Procedures for hybridization and washing the membrane were as previously reported^56^. The primer sequences are listed in Supplementary Table 2. For western blotting, a specific amino acid sequence from the same region of *SSN1* and *SSN2* (residues 272–286 DHRKGGRWDENKKEK) was used as the antigen through commercial synthesis; the antibody was prepared by Neweast Bioscience (Wuhan, China). Anti-histone 3 (ab1791, Rabbit polyclonal to Histone H3—ChIP Grade, ABcam, San Francisco, CA) was used as an endogenous standard. Total protein was extracted from WT, *SSN*-RNAi and overexpressing seedlings using the extraction buffer. Protein concentrations were determined using the Bradford assay. Western blot experiments were performed as described previously^58^. The primary antibody was diluted at 1:5,000 for SSN probing or 1:10,000 for histone probing. A secondary anti-rabbit horseradish peroxidase labelled IgG was subsequently added. Signal of protein was detected using the Pierce Supersignal West Pico kit (Thermo Scientific, Rockford, IL).

### Transient expression and subcellular localization

Full-length *SSN1* cDNA without a stop codon was isolated using the primers forward, 5′-GGGGACAAGTTTGTACAAAAAAGCAGGCTTCATGGATCTTCTTGATTTCTCCACT-3′, and reverse, 5′-GGGGACCACTTTGTACAAGAAAGCTGGGTTGTTATAGAGCTCAGGAGCAAGGC-3′, carrying the attB adapter sites and recombined into the vector pMDC83 (ref. 59) by BP and LR recombination reactions (Gateway Technology); the resulting construct consisted of *SSN1* fused to the green fluorescent protein N terminus controlled by the CaMV 35S promoter. An RFP fusion with the chaperone-binding protein BiP was used as an endoplasmic reticulum marker protein. The fusion constructs were introduced into *Nicotiana benthamiana* protoplasts isolated from seedling leaves by polyethylene glycol/calcium-mediated transformation^60^. Fluorescence microscopy was performed using a Leica TCS SP2 confocal spectral microscope (Leica, Heidelberg, Germany).

### Lipid analysis

WT and transgenic plant cotyledons were quickly immersed in 3.0 ml 75 °C isopropanol with 0.01% butylated hydroxytoluene for 15 min. The mixture was vortexed after adding 1.5 ml chloroform and 0.6 ml water; it was then agitated (on shaking incubator) at room temperature for 1 h. The lipid extracts were transferred to glass screw-cap (Teflon-lined) tubes. Next, 4.0 ml chloroform/methanol (2:1) with 0.01% butylated hydroxytoluene was added before shaking for 30 min. The above procedure was repeated for all samples until the leaves of each sample became white. The process typically required approximately five extractions, including the isopropanol extraction. KCl (1 M, 1.0 ml) was added to the combined extract, the sample mixed by vortexing or shaking, then centrifuged to break up the phases and the upper phase was discarded. Water (2.0 ml) was added, vortexed or shaken, centrifuged and the upper phase was discarded. Extracted leaves were dried at 80 °C in an oven overnight for dry weight determination to four decimal places (0.0000, g). Samples were analysed by the KLRC (Kansas Lipidomics Research Center). The free fatty acids were analyzed in the (Applied Biosystems) API4000 triple quad mass spec using the negative MS1 scan mode. The source temperature was 100 °C, the desolvation temperature was 250 °C, 2.8 kV was applied to the electrospray capillary, the cone energy was 40 V and argon was used as the collision gas at 1.7 e^−3^ mBar as measured on the gauge in the collision cell line.

### Phytohormone and oxylipin quantitation

For JA analysis, the samples (100–200 mg) were extracted twice with 80% cold methanol (v/v) overnight at 4 °C. To each sample was added 10 ng (±)-9,10-dihydro-JA (Olchemim) as an internal standard. The combined extract was evaporated to the aqueous phase with N_2_, dissolved in 0.4 ml methanol and then filtered using a syringe-facilitated filter (Nylon 66; Jin Teng Experiment Equipment Co., Tianjin, China). The samples were stored at −80 °C before the measurements. The JA levels were quantified using an HPLC-MS/MS system (AB SCIEX Triple Quad 5500 LC/MS/MS) with JA (Sigma) as the external standards. For JA-Ile and SA analysis, the JA-Ile and SA levels were quantified with JA-Ile and SA (Sigma) as the external standards. To estimate the oxylipins levels, the samples were obtained and extracted as described for phytohormone analysis. The supernatants were analysed using an HPLC-MS/MS system (AB SCIEX Triple Quad 5500 LC/MS/MS system) with 9- or 13-HPOD, 9- or 13-HPOT, 9- or 13-HOD, 9- or 13-HOT or 9- or 13-KOD (Cayman Chemical Co) as the external standard.

## Author contributions

X.Z. and L.Z. conceived and designed the experiments; L.X. constructed the suppression subtractive hybridization cDNA library; D.Y. annotated the RNA-Seq data; L.S. performed the experiments, analysed the data and wrote the paper and X.Z. and L.M. corrected the manuscript. All of the authors discussed the results and commented on the manuscript.

## Additional information

**Accession codes:** cDNA sequences have been deposited in the NCBI GenBank database with the following accession codes: GhCYP82D1 (SSN1), KJ704109; cDNA of GhCYP82D2 (SSN2), KJ704110; cDNA of GhCYP82D3 (SSN3), KJ704111. Raw RNA-seq data have been deposited in the Sequence Read Archive (SRA) under accession codes SRR1569164.

**How to cite this article**: Sun, L. *et al.* Cotton cytochrome P450 CYP82D regulates systemic cell death by modulating the octadecanoid pathway. *Nat. Commun.* 5:5372 doi: 10.1038/ncomms6372 (2014).

## Supplementary Material

Supplementary Information Supplementary Figures 1-16, Supplementary Tables 1-2.

## Figures and Tables

**Table 1:** Identification by Illumina sequencing of genes differentially expressed in WT and Ri15.

**Gene ID**	**Description**	**Ratio**
zhu1_Ghi#S42295371	LOX1, lipoxygenase 1	1.70
zhu1_Ghi#S30014770	LOX2, lipoxygenase 2	1.87
zhu1_Ghi#S42334867	LOX5, domain-containing lipoxygenase family protein	1.51
zhu1_Ghi#S28658229	AOS, allene oxide synthase	2.09
zhu1_Ghi#S42308858	AOC4, allene oxide cyclase 4	1.68
zhu1_Ghi#S42360391	JAZ1, jasmonate-zim-domain protein 1	1.49
zhu1_Ghi#S42304649	JAZ3, jasmonate-zim-domain protein 3	1.41
zhu1_Ghi#S42326912	JAZ10, jasmonate-zim-domain protein 10	1.69
zhu1_Ghi#S33804033	MYC2-like, bHLH DNA-binding superfamily protein	1.26
zhu1_Ghi#S28670457	WRKY70, WRKY DNA-binding protein 70	−1.58
zhu1_Ghi#S28671572	WRKY51, WRKY DNA-binding protein 51	−1.58
zhu1_Ghi#S33814007	WRKY50, WRKY DNA-binding protein 50	−1.67
zhu1_Ghi#S42359955	WRKY46, WRKY DNA-binding protein 46	−1.30

The genes in this list were selected from the Illumina sequencing for further characterization. Shown are locus designations, descriptions of putatively encoded proteins and differential expression ratios (log2) between WT and Ri15 in roots of 6-day-old seedlings on the medium.
