# Supplementary material for: Cotton cytochrome P450 CYP82D regulates systemic cell death by modulating the octadecanoid pathway
Source: Nat Commun. 2014 Nov 5;5:5372. doi: 10.1038/ncomms6372 (PMC4241986; doi:10.1038/ncomms6372)
Supplement: Supplementary Information — Supplementary Figures 1-16, Supplementary Tables 1-2. [file ncomms6372-s1.pdf]

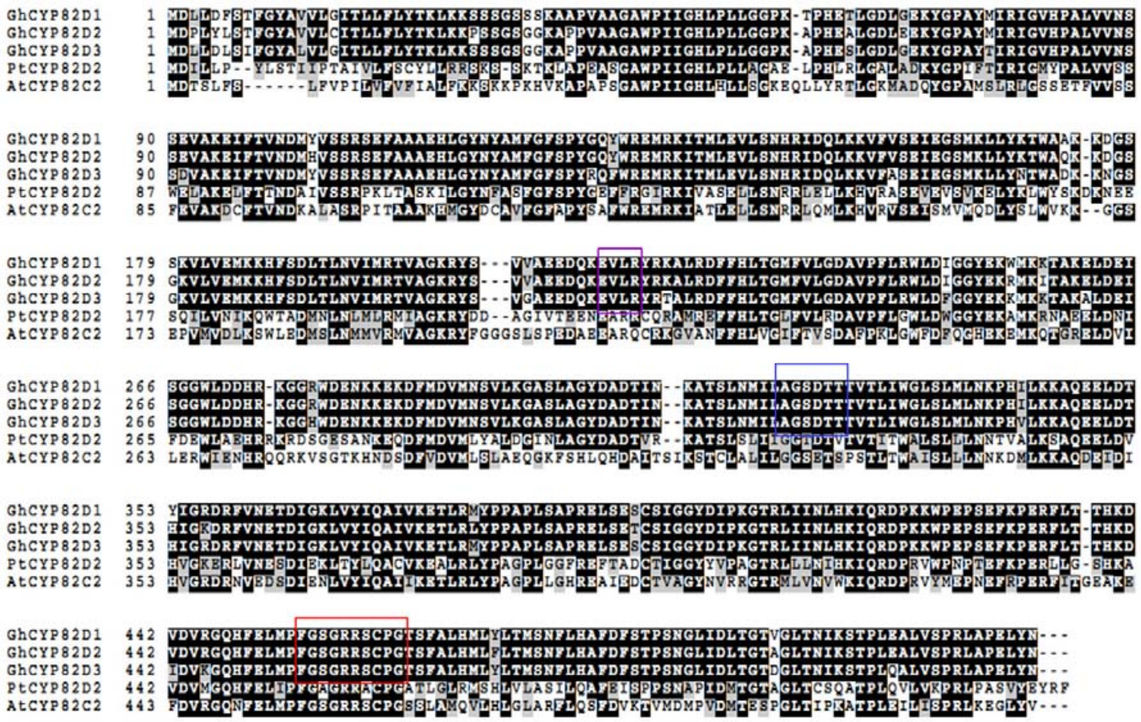

Supplementary Fig. 1. Comparison of amino acid sequences for GhCYP82D1 (SSN1), GhCYP82D*2* (SSN2), and GhCYP82D3 (SSN3) with PtCYP82D2 and AtCYP82C2. The helix K domain is marked by the purple box; the helix I domain, by the blue box; and the heme-binding domain, by the red box.

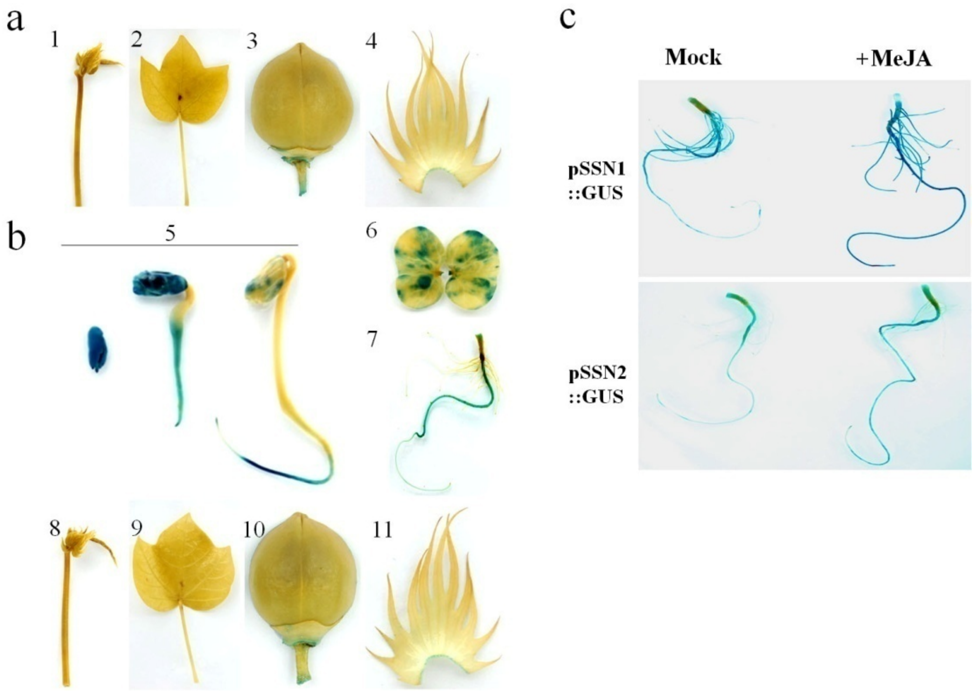

Supplementary Fig. 2. GUS detection. (a) SSN1 promoter::GUS fusion expression patterns in transgenic cotton. GUS staining was undetectable in the branch (1), mature leaf (2), cotton boll (3) and bract (4). (b) SSN2 promoter::GUS fusion expression patterns in transgenic cotton. GUS staining was shown in the germinating seeds (5), cotyledon (6) and root (7) of young seedlings but was undetectable in the branch (8), mature leaf (9), cotton boll (10) and bract (11). (c) MeJA-induced pSSN1::GUS and pSSN2::GUS expression in the root. pSSN1::GUS and pSSN2::GUS seedlings (9-day-old) were exposed to 100 μM MeJA for 2 hours and stained overnight.

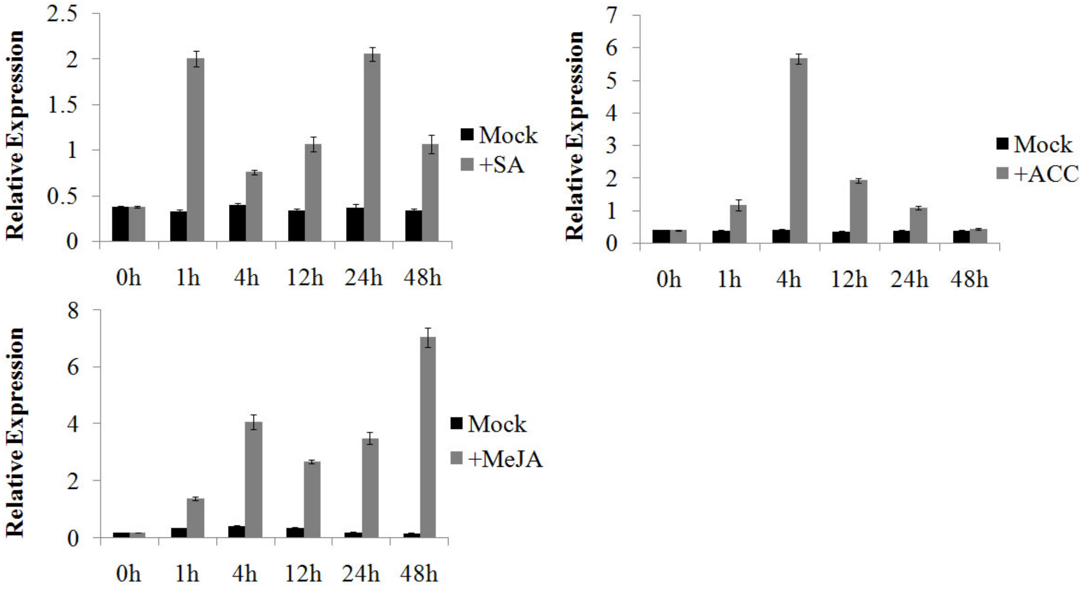

Supplementary Fig. 3. *SSN* responses to phytohormone MeJA, SA and ACC treatments in WT cotton YZ1. The plants were pre-treated with Hoagland's; MeJA, ACC and SA were then added to the solution to the final concentrations of 50 μM, 5 μM and 0.5 mM, respectively. qRT-PCR was performed at 0, 1, 4, 12, 24 and 48 h after treatment. Error bars indicate SD from four technical replicates. Gene expression levels were normalised to *UBQ7*.

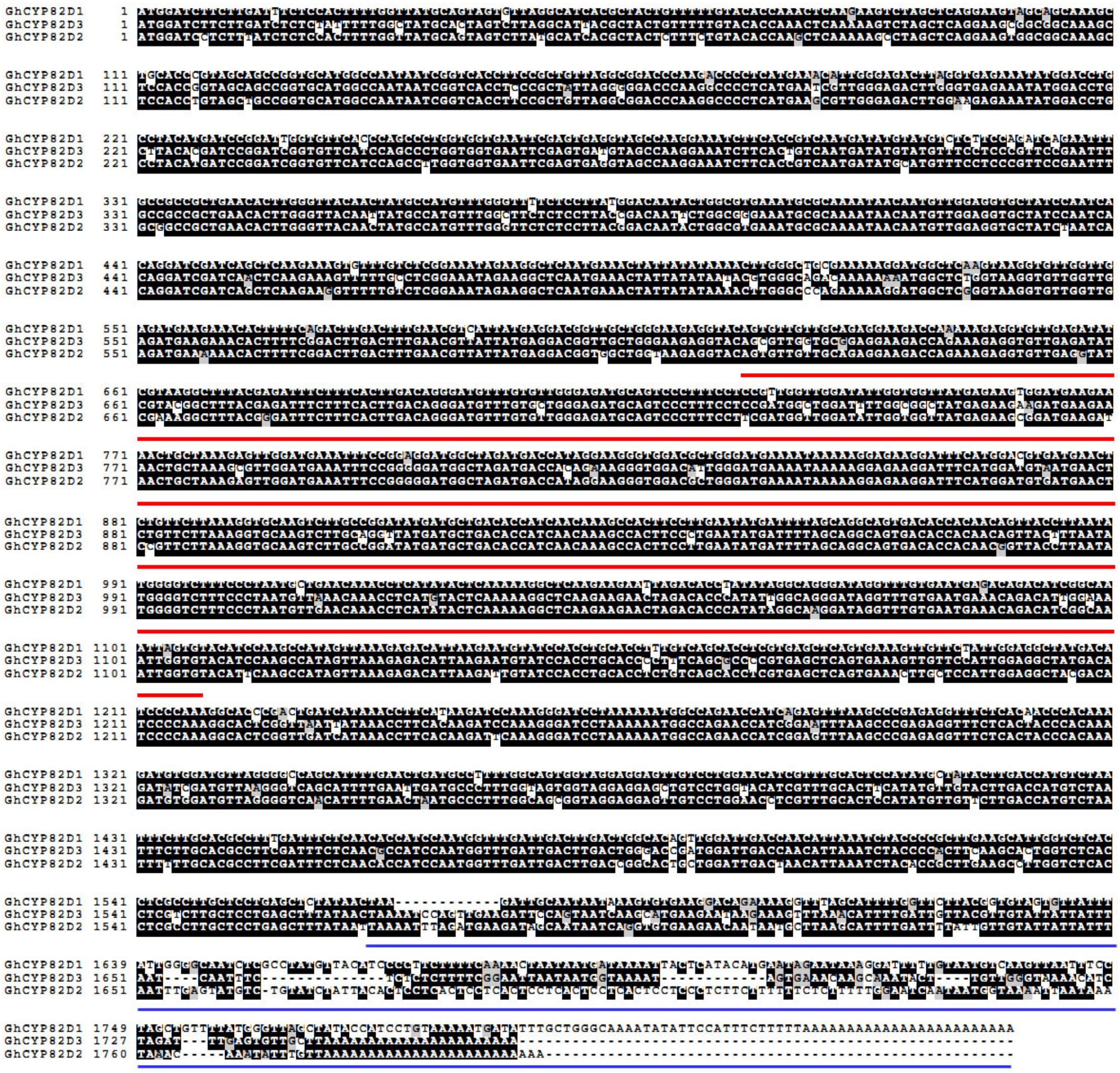

Supplementary Fig. 4. Comparison of the nucleotide sequences for the genes *GhCYP82D1* (*SSN1*), *GhCYP82D2* (*SSN2*) and *GhCYP82D3* (*SSN3*) to show their highly similar ORFs. The 3'UTR sequences are marked by blue lines. The RNAi vector for the conserved region was constructed using the nucleotide sequence marked with red lines.

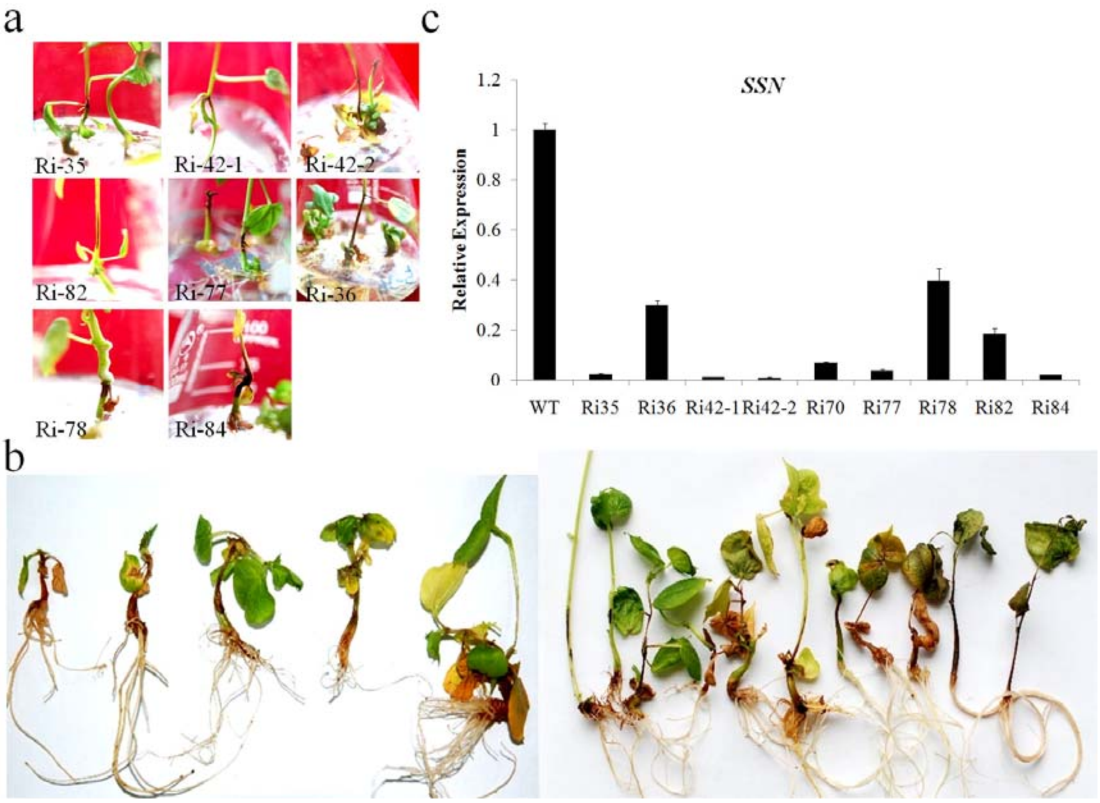

Supplementary Fig. 5. The *SSN*-RNAi T0 seedling lesion phenotype and *SSN* expression level for the transgenic plants analysed using qRT-PCR. (**a**) The lesion phenotype appeared on regenerated seedling stems. (**b**) A large number of transgenic plants died in plant regeneration medium before being transplanted to the soil. (**c**) The qRT-PCR analysis showed that *SSN* expression was downregulated in transgenic seedlings. Error bars indicate SD from four technical replicates. The gene expression levels were normalised to *UBQ7*.

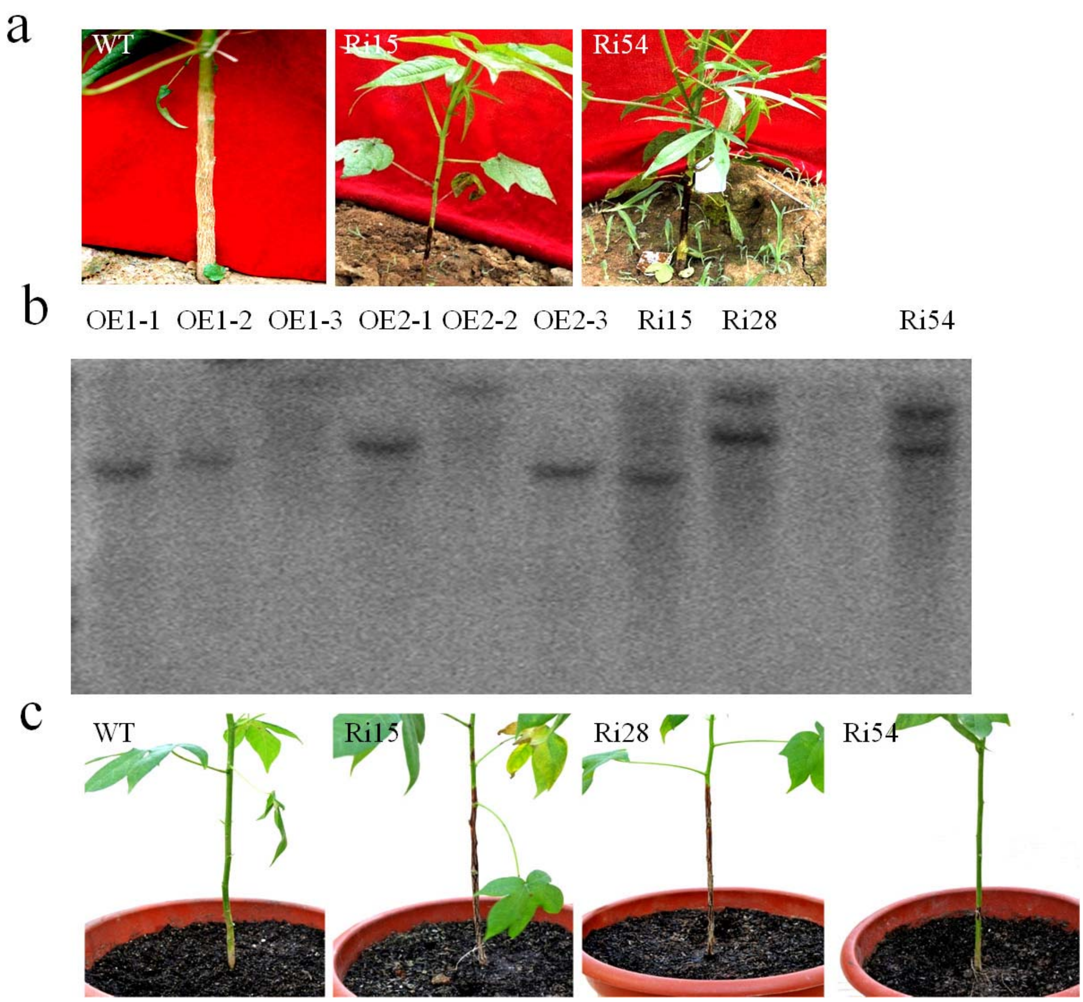

Supplementary Fig. 6. Phenotypic observations and Southern blot analysis**.** (**a**) The lesion phenotype of *SSN*-RNAi T0 lines in the field. (**b**) Southern blot analysis for transgenic plants. The probe was *NPTII*. The plants were treated as separate transgenic events in the following analysis. (**c**) The lesion phenotype of *SSN*-RNAi offspring cultured in the green house.

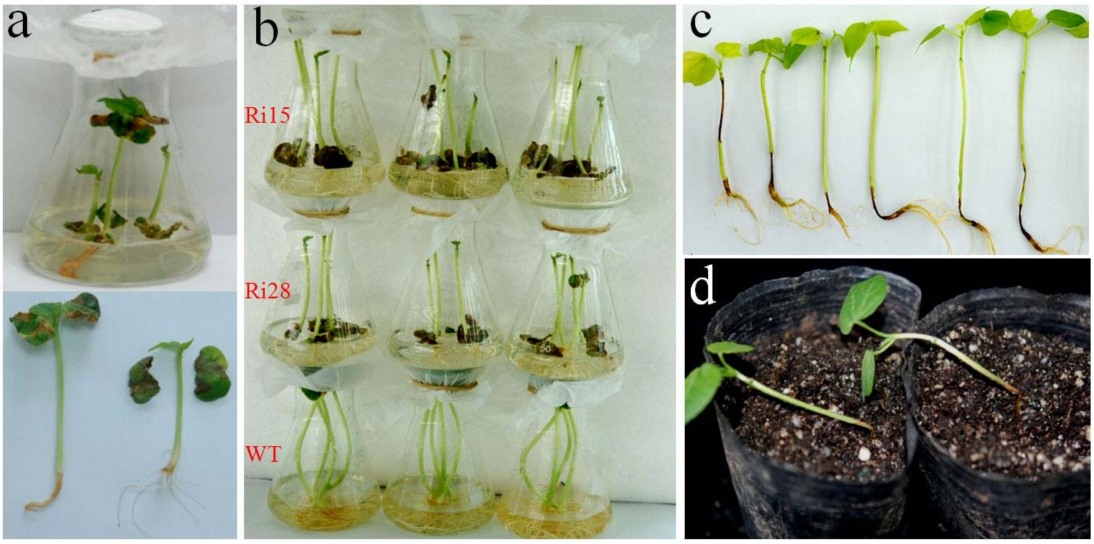

Supplementary Fig. 7. The lesion phenotype of *SSN*-RNAi offspring. (**a**) Cotyledons from the Ri15 T1 line showed a lesion phenotype and abscised earlier on media containing kanamycin (plants that develop roots are transformants on this medium). (**b**) Cotyledons from the T3 lines showed a lesion phenotype and abscised earlier on the media. (**c**, **d**) The transgenic offspring stems showed a lesion phenotype approximately two or three weeks not only under Hoagland's solution conditions but also under small-pot soil culture conditions.

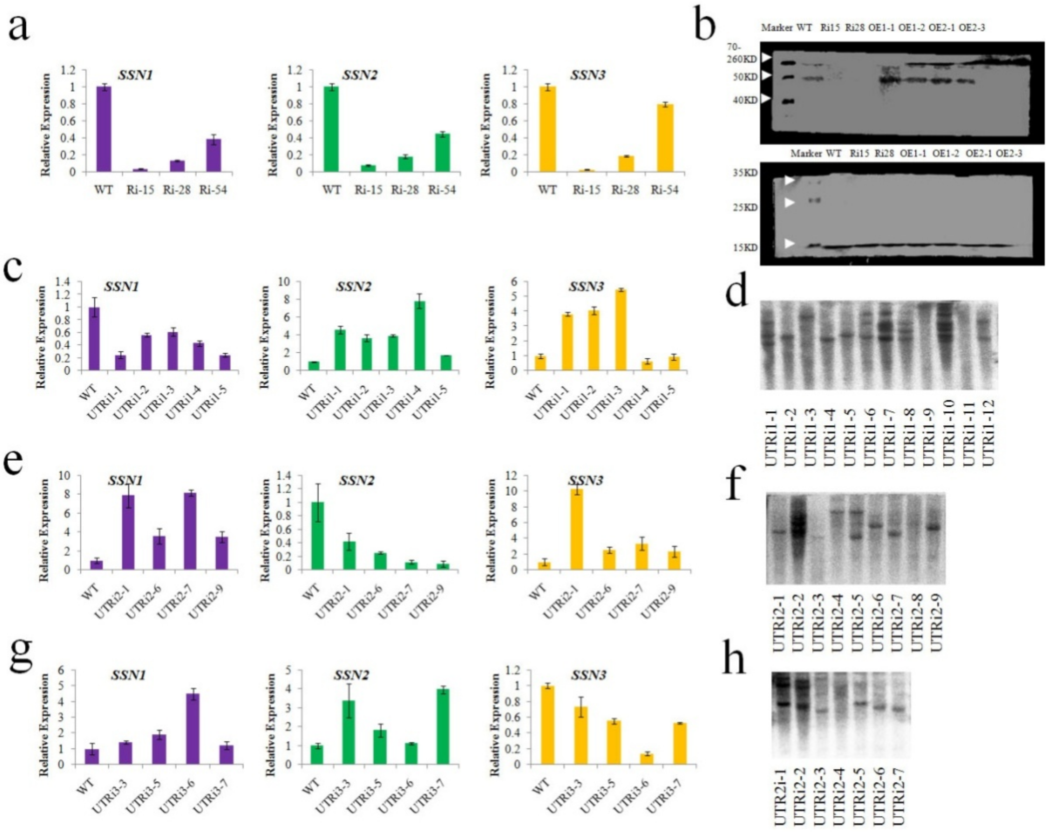

Supplementary Fig. 8. 3’-UTR specific silencing analysis of the *SSN* gene family. (**a**) qRT-PCR analysis of *SSN1*, *SSN2* and *SSN3* expression in *SSN* conserved-region silenced lines. The *SSN1*, *SSN2* and *SSN3* expression levels in each line were calculated relative to the WT plants. The RNA was extracted from roots of 6-day-old homozygous seedlings grown on medium. (**b**) Western blotting revealed different expression levels among the transgenic and WT plants leaves. Antibodies were prepared using the same SSN1 and SSN2 protein amino acid sequences as the probes; anti-histone was used as a control. Ri: *SSN*-RNAi plants; OE1: *SSN1*-overexpression plants; OE2: *SSN2*-overexpression plants. (**c, d**) qRT-PCR analysis of *SSN1*, *SSN2* and *SSN3* expression in UTRi1 (*SSN1*) silenced lines. For the Southern blot analysis, the transgenic plants were probed by *NPTII*. (**e, f**) qRT-PCR analysis of *SSN1*, *SSN2* and *SSN3* expression in UTRi2 (*SSN2*) silenced lines. For the Southern blot analysis, the

transgenic plants were probed by *NPTII*. (**g, h**) qRT-PCR analysis of *SSN1*, *SSN2* and *SSN3* expression in UTRi3 (*SSN3*) silenced lines. For the Southern blot analysis, the transgenic plants were probed by *NPTII*. Error bars indicate SD from four technical replicates. The gene expression levels were normalised to *UBQ7*.

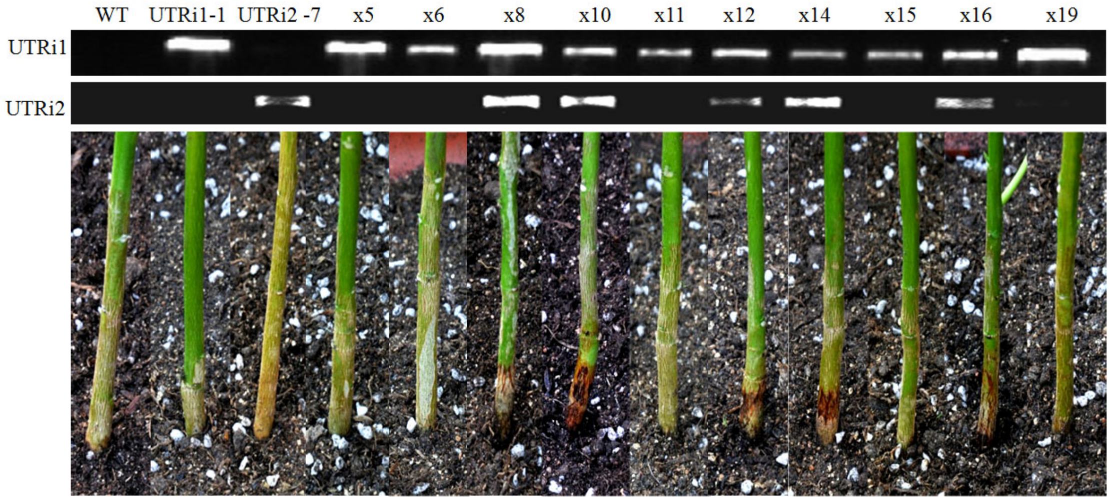

Supplementary Fig. 9. F1 plants from crossing a homozygous UTRi1 (*SSN1*) silenced plant with a heterozygous UTRi2 (*SSN2*) silenced plant; one gene-specific primer (from the 3'-UTR of *SSN1* or 3'-UTR of *SSN2*) and one vector primer (from a 35S promoter) were used to amplify and examine the transgene segment in F1 plants. Plants with both amplifications showed the lesion mimic phenotype, but plants with only one amplification did not show a lesion phenotype.

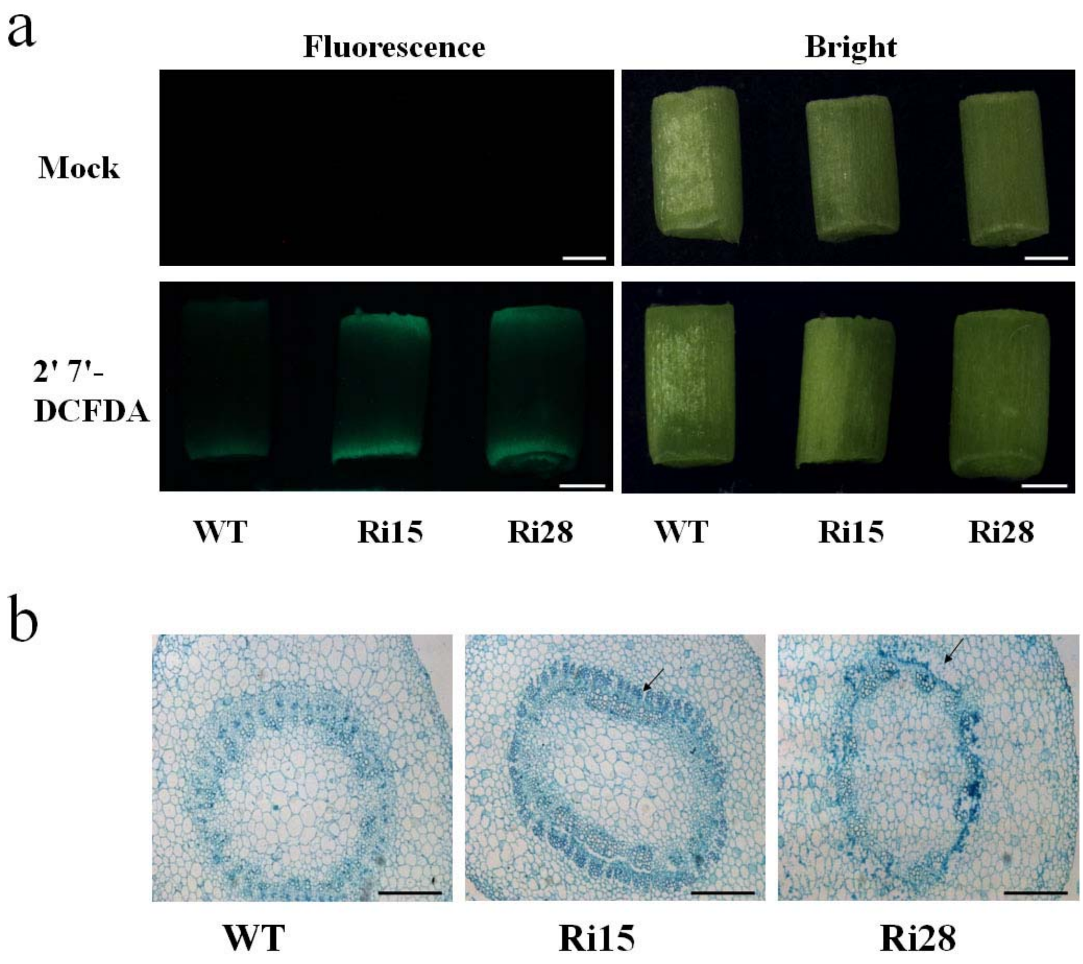

Supplementary Fig. 10. ROS detection by 2',7'-DCFDA staining in transverse section of hypocotyl segments of 9 day-old seedlings. (a) Fluorescence and bright field image of ROS in stems of *SSN*-RNAi and wild-type lines by 2′,7′-DCFDA staining. Bars = 1 mm. (b) Transverse section of the 9 day-old stem segments. Bar = 40 µm. The cells of vascular bundles in *SSN*-RNAi showed necrosis and were collapsed.

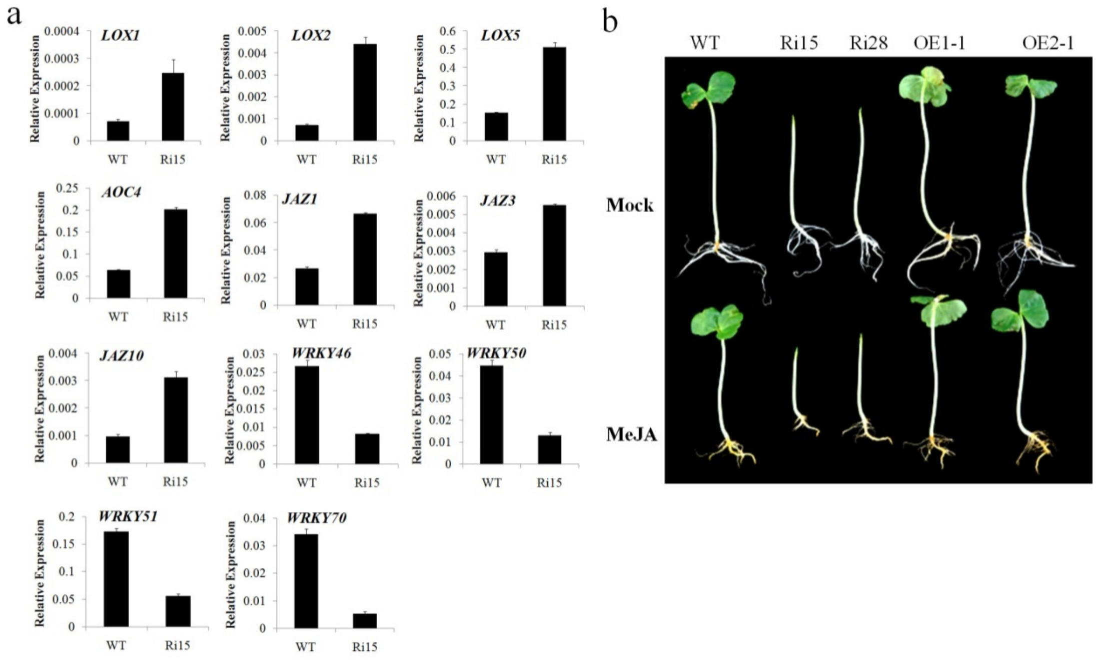

Supplementary Fig. 11. Verification of the RNA-Seq data in *SSN*-RNAi plants. (**a**) Selected genes based on the JA synthesis and signal transduction pathways from the RNA-Seq data were verified using qRT-PCR. RNA was extracted from 6 day-old seedling roots. The values are the means ± SD for three biological replicates. (**b**) The morphological phenotype of WT and overexpression seedlings showed no difference in sensitivity in JA-induced growth inhibition when grown for 7 days on media containing MeJA (1 μM). The *SSN*-RNAi seedling cotyledons had already lost cotyledons.

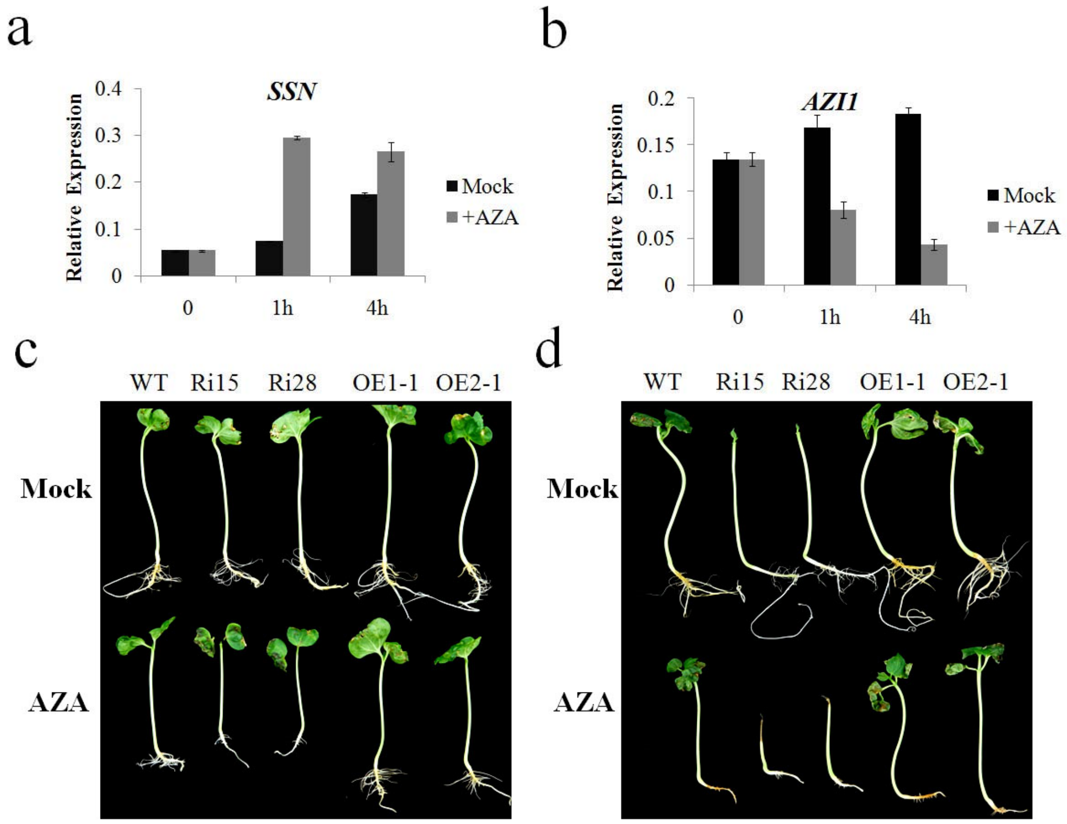

Supplementary Fig. 12. Gene expression and phenotype changes in cotton seedlings in response to the AZA treatments. (**a**) *SSN* responses to the AZA treatments in WT plants. RNA was extracted from 3 week-old seedling roots at different intervals after AZA (1mM) application. (**b**) Expression of the AZA-inducible gene *AZI1* in WT plants after the AZA application. Data (**a**, **b**) are presented as the means ± SD collected from four technical replicates. (**c**) Morphological phenotypes for WT and transgenic lines treated with AZA (0.2 mM) in the medium. 1.5 day-old plants were subjected to different treatments and photographed 5 days after treatment. Many AZA-treated *SSN*-RNAi plants showed typical lesion mimic symptoms on the cotyledons. (**d**) Plants treated with AZA (0.5 mM) in the medium. 1.5 day-old plants were subjected to different treatments and photographed 10 days after treatment. High concentrations of AZA treatment enhanced the stem lesion mimic phenotype.

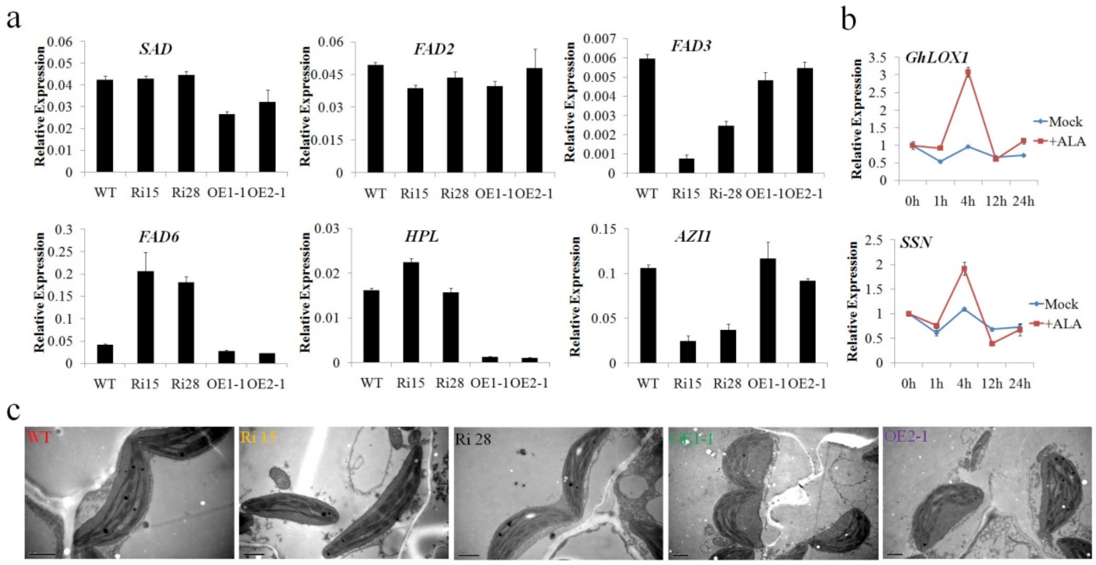

Supplementary Fig. 13. SSN modulates fatty acids metabolism. (**a**) Key genes selected from the octadecanoid pathway showed significantly different expression levels in *SSN*-RNAi and OE lines. SAD, stearoyl-acyl carrier protein desaturase; FAD2, ω-6 fatty-acid desaturase; FAD3, ω-3 fatty-acid desaturase; FAD6, ω-6 fatty acid desaturase; HPL, hydro-peroxide lyase; and AZI1, azelaic acid induced 1. RNA was extracted from roots of 6-day-old seedlings. (**b**) The expression pattern analysis for *SSN* in response to the linolenic acid (ALA) treatments in cotton cotyledons using qRT-PCR. RNA was extracted from seedlings at different intervals after an ALA (10 mM) application. *GhLOX1* and *SSN* were significantly upregulated in the cotyledon following ALA treatment. Data (**a**, **b**) are presented as the means ± SD collected from four technical replicates. (**c**) TEM micrographs of cotyledon cells from WT and transgenic plants showing that chloroplast development was retarded in the overexpression lines. Scale bars = 1 μm.

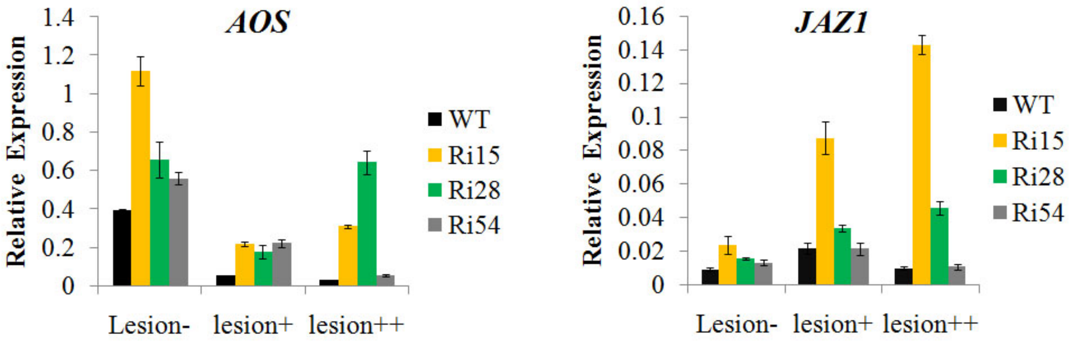

Supplementary Fig. 14. *AOS* and *JAZ1* transcript levels at the different lesion formation stages shown in **Fig. 6a**. Genes involved in jasmonate biosynthesis were constitutively activated. Total RNA was extracted from cotyledons as shown in **Fig. 6a** and subjected to qRT-PCR analyses. The values are the means ± SD of three biological replicates. Gene expression levels were normalised to *UBQ7*.

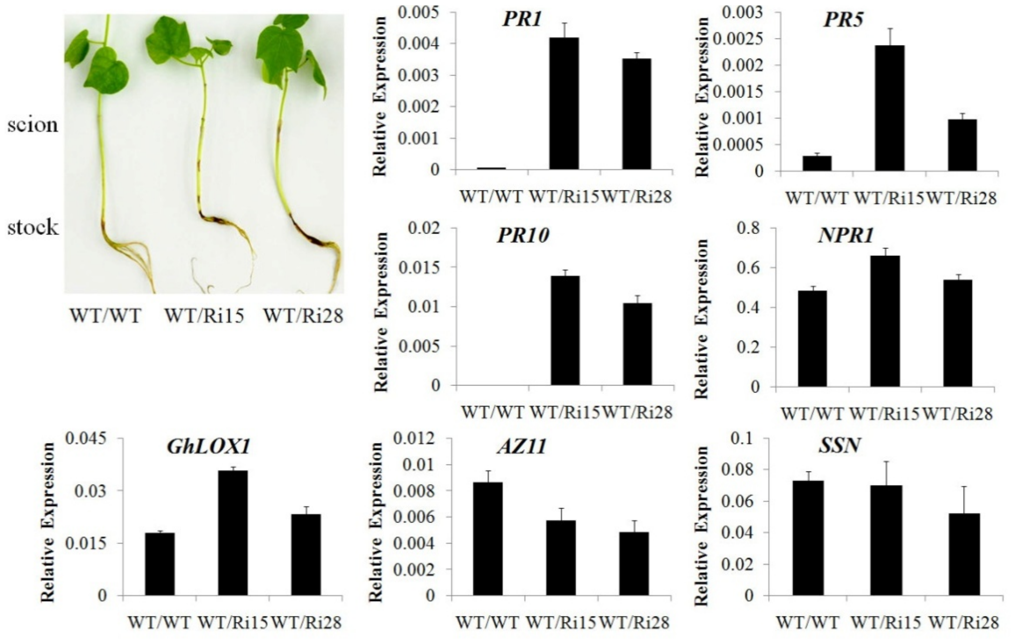

Supplementary Fig. 15. Grafting studies and gene expression in grafted scion leaves. The SAR marker genes were constitutively activated. WT/WT, WT/Ri15 and WT/Ri28 indicate WT scions grafted onto wild-type or *SSN*-RNAi rootstocks. Total RNA was extracted from leaves of 3 week-old plants after grafting and subjected to qRT-PCR analyses. The values are the means ± SD for three biological replicates. Transcript levels of each gene were normalised to *UBQ7*.

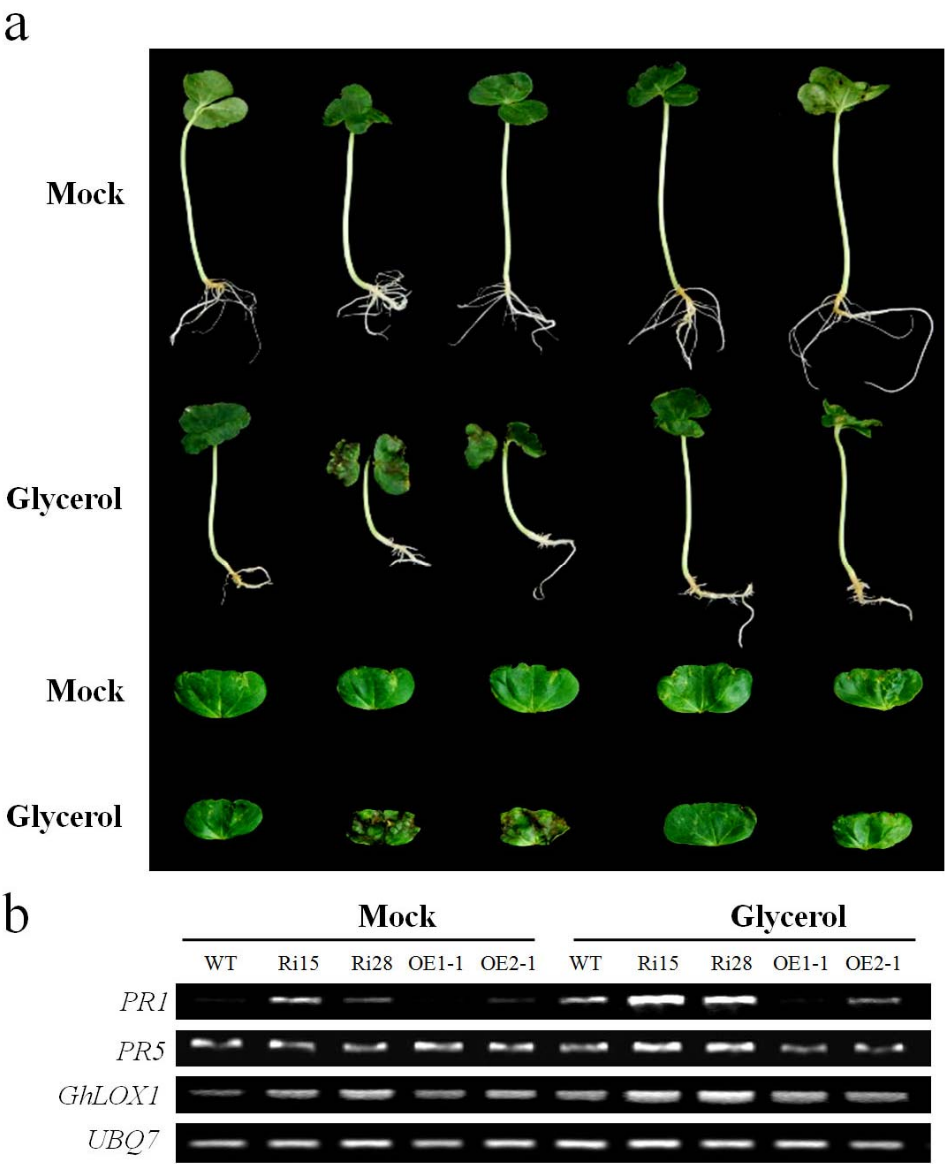

Supplementary Fig. 16. Morphological phenotype of the WT and transgenic seedlings treated by glycerol on the media. (**a**) Silenced plants showed more sensitivity to glycerol-mediated growth inhibition and exhibited accelerated and aggravated HR-like necrosis for the seedlings grown for 5 days on media containing glycerol (0.5%). (**b**) RT-PCR analysis of the marker genes associated with HR in transgenic seedling cotyledons grown for 3 days on glycerol (0.5%) media. The expression levels of the three marker genes were all stronger in *SSN*-RNAi plants. The cotton *UBQ7* gene was amplified as a control.

Supplementary Table 1 Genes differentially expressed between WT and Ri15in roots as determined using RNA-Seq.

GeneID	log2 Ratio(WT/*SSN*-RNAi)	P-value	FDR	Description (WT/*SSN-RNAi*)
zhu1_Ghi#S28697621	3.316571447	3.18E-06	6.04E-05	AT1G26770.2 | Symbols: ATEXPA10, AT-EXP10, ATEXP10, ATHEXP ALPHA 1.1, EXP10,EXPA10 | expansin A10 | chr1:9259775-9260792 FORWARDLENGTH=259
zhu1_Ghi#S33808000	2.38963491	1.60E-07	3.86E-06	AT1G49820.1 | Symbols: ATMTK, MTK | S-methyl-5-thioribose kinase |chr1:18443005-18444907 FORWARD LENGTH=420
zhu1_Ghi#S28658229	2.088465375	0	0	AT5G42650.1 | Symbols: AOS, CYP74A, DDE2 | allene oxide synthase |chr5:17097803-17099359 REVERSE LENGTH=518
zhu1_Ghi#S42301433	1.980362433	1.27E-13	8.05E-12	AT5G42650.1 | Symbols: AOS, CYP74A, DDE2 | allene oxide synthase |chr5:17097803-17099359 REVERSE LENGTH=518
zhu1_Ghi#S28674097	1.97459741	0	0	AT3G11340.1 | Symbols: | UDP-Glycosyltransferase superfamily protein |chr3:3556728-3558149 FORWARD LENGTH=447
zhu1_Ghi#S30014770	1.866600158	5.57E-08	1.46E-06	AT3G45140.1 | Symbols: LOX2, ATLOX2 | lipoxygenase 2 | chr3:16525437-16529233FORWARD LENGTH=896
zhu1_Ghi#S33838870	1.778385717	1.21E-10	4.33E-09	AT5G22580.1 | Symbols: | Stress responsive A/B Barrel Domain |chr5:7502709-7503137 FORWARD LENGTH=111
zhu1_Ghi#S42283807	1.754471596	0	0	AT1G05010.1 | Symbols: EFE, ACO4, EAT1 | ethylene-forming enzyme |chr1:1431419-1432695 REVERSE LENGTH=323
zhu1_Ghi#S42295371	1.699641979	3.73E-13	2.25E-11	AT1G55020.1 | Symbols: LOX1, ATLOX1 | lipoxygenase 1 | chr1:20525798-20530143FORWARD LENGTH=859
zhu1_Ghi#S42326912	1.691880639	1.70E-12	8.82E-11	AT5G13220.1 | Symbols: JAZ10, TIFY9, JAS1 | jasmonate-zim-domain protein 10 |chr5:4219001-4220502 FORWARD LENGTH=197
zhu1_Ghi#S42308858	1.682416659	0	0	AT1G13280.1 | Symbols: AOC4 | allene oxide cyclase 4 | chr1:4547624-4548552FORWARD LENGTH=254

zhu1_Ghi#S33825599	1.679141527	0	0	NoHits
zhu1_Ghi#S42301072	1.660135446	0	0	AT2G02990.1 | Symbols: RNS1, ATRNS1 | ribonuclease 1 | chr2:873714-874667FORWARD LENGTH=230
zhu1_Ghi#S28640612	1.656082511	1.98E-12	1.02E-10	NoHits
zhu1_Ghi#S42305390	1.64787253	6.75E-14	4.38E-12	AT1G68560.1 | Symbols: ATXYL1, XYL1, TRG1 | alpha-xylosidase 1 |chr1:25734435-25737897 REVERSE LENGTH=915
zhu1_Ghi#S33832915	1.631926888	2.61E-07	6.08E-06	AT3G61460.1 | Symbols: BRH1 | brassinosteroid-responsive RING-H2 |chr3:22741701-22742213 REVERSE LENGTH=170
zhu1_Ghi#S30013426	1.626526901	1.71E-09	5.35E-08	AT1G09815.1 | Symbols: POLD4 | polymerase delta 4 | chr1:3189460-3190050FORWARD LENGTH=124
zhu1_Ghi#S42321096	1.590243723	7.54E-13	4.21E-11	NoHits
zhu1_Ghi#S42300243	1.555443803	4.59E-07	1.03E-05	AT3G12110.1 | Symbols: ACT11 | actin-11 | chr3:3858116-3859609 FORWARDLENGTH=377
zhu1_Ghi#S42334867	1.5133129	3.61E-12	1.71E-10	AT3G22400.1 | Symbols: LOX5 | PLAT/LH2 domain-containing lipoxygenase familyprotein | chr3:7927011-7931167 FORWARD LENGTH=886
zhu1_Ghi#S42360391	1.490465351	7.11E-08	1.83E-06	AT1G19180.1 | Symbols: JAZ1, TIFY10A | jasmonate-zim-domain protein 1 |chr1:6622312-6623271 FORWARD LENGTH=253
zhu1_Ghi#S28663402	1.477726239	3.78E-07	8.57E-06	NoHits
zhu1_Ghi#S29997408	1.477359654	8.88E-13	4.84E-11	NoHits
zhu1_Ghi#S42360575	1.467637422	0	0	AT4G15560.1 | Symbols: CLA1, DEF, CLA, DXS, DXPS2 | Deoxyxylulose-5-phosphatesynthase | chr4:8884218-8887254 FORWARD LENGTH=717
zhu1_Ghi#S33809128	1.424657195	0	0	AT1G78550.1 | Symbols: | 2-oxoglutarate (2OG) and Fe(II)-dependent oxygenasesuperfamily protein | chr1:29545031-29546360 REVERSELENGTH=356
zhu1_Ghi#S42305326	1.414990484	1.61E-09	5.06E-08	AT5G12010.1 | Symbols: | unknown protein; INVOLVED IN: response to salt

				stress;LOCATED IN: chloroplast, plasma membrane, membrane;EXPRESSED IN: 23 plant structures; EXPRESSED DURING: 13growth stages; BEST Arabidopsis thaliana protein matchis: unknown protein (TAIR:AT4G29780.1); Has 1807 Blasthits to 1807 proteins in 277 species: Archae - 0;Bacteria - 0; Metazoa - 736; Fungi - 347; Plants - 385;Viruses - 0; Other Eukaryotes - 339 (source: NCBI BLink).| chr5:3877975-3879483 REVERSE LENGTH=502
zhu1_Ghi#S42304649	1.409312394	0	0	AT3G17860.3 | Symbols: JAZ3, JAI3, TIFY6B | jasmonate-zim-domain protein 3 |chr3:6121143-6122691 FORWARD LENGTH=319
zhu1_Ghi#S33813361	1.401962939	0	0	AT3G04090.1 | Symbols: SIP1;1, SIP1A | small and basic intrinsic protein 1A |chr3:1072340-1074031 REVERSE LENGTH=240
zhu1_Ghi#S42359795	1.367075284	1.06E-12	5.71E-11	AT2G41480.1 | Symbols: | Peroxidase superfamily protein |chr2:17296986-17298729 REVERSE LENGTH=341
zhu1_Ghi#S33824240	1.348992925	2.83E-06	5.45E-05	AT1G78830.1 | Symbols: | Curculin-like (mannose-binding) lectin family protein| chr1:29637141-29638508 REVERSE LENGTH=455
zhu1_Ghi#S42310950	1.348992925	1.08E-09	3.49E-08	AT4G28940.1 | Symbols: | Phosphorylase superfamily protein |chr4:14274414-14276920 FORWARD LENGTH=348
zhu1_Ghi#S28675209	1.316078303	1.30E-13	8.21E-12	AT5G57560.1 | Symbols: TCH4, XTH22 | Xyloglucan endotransglucosylase/hydrolasefamily protein | chr5:23307296-23308235 REVERSELENGTH=284
zhu1_Ghi#S31153413	1.312819313	4.92E-13	2.90E-11	AT4G11650.1 | Symbols: ATOSM34, OSM34 | osmotin 34 | chr4:7025127-7026113REVERSE LENGTH=244
zhu1_Ghi#S42304188	1.312095013	1.35E-12	7.09E-11	AT1G05010.1 | Symbols: EFE, ACO4, EAT1 | ethylene-forming enzyme |chr1:1431419-1432695 REVERSE LENGTH=323
zhu1_Ghi#S33811615	1.31151822	5.79E-07	1.28E-05	AT4G39300.1 | Symbols: | unknown protein; FUNCTIONS IN: molecular_functionunknown; INVOLVED IN: biological_process unknown;LOCATED IN: chloroplast; EXPRESSED IN: 22 plantstructures;

				EXPRESSED DURING: 13 growth stages; Has30201 Blast hits to 17322 proteins in 780 species:Archae - 12; Bacteria - 1396; Metazoa - 17338; Fungi -3422; Plants - 5037; Viruses - 0; Other Eukaryotes -2996 (source: NCBI BLink). | chr4:18286835-18287823FORWARD LENGTH=96
zhu1_Ghi#S42287938	1.276236583	1.41E-09	4.47E-08	NoHits
zhu1_Ghi#S33804033	1.262442893	1.01E-11	4.30E-10	AT4G00870.1 | Symbols: | basic helix-loop-helix (bHLH) DNA-binding superfamilyprotein | chr4:362230-363639 REVERSE LENGTH=423
zhu1_Ghi#S42282252	1.255883521	3.69E-14	4.30E-10	AT5G13980.2 | Symbols: | Glycosyl hydrolase family 38 protein |chr5:4508626-4514334 FORWARD LENGTH=1024
zhu1_Ghi#S42281949	1.243101757	1.42E-09	4.30E-10	AT5G54160.1 | Symbols: ATOMT1, OMT1 | O-methyltransferase 1 |chr5:21982075-21984167 FORWARD LENGTH=363
zhu1_Ghi#S42305093	1.229693997	4.08E-09	4.30E-10	AT3G02040.1 | Symbols: SRG3 | senescence-related gene 3 | chr3:348505-349909REVERSE LENGTH=361
zhu1_Ghi#S28641947	1.229572655	4.62E-13	4.30E-10	AT3G09640.2 | Symbols: APX2, APX1B | ascorbate peroxidase 2 |chr3:2956301-2958163 FORWARD LENGTH=251
zhu1_Ghi#S42359305	1.197348399	8.05E-13	4.30E-10	AT1G17020.1 | Symbols: SRG1, ATSRG1 | senescence-related gene 1 |chr1:5820258-5821741 FORWARD LENGTH=358
zhu1_Ghi#S42328464	1.195009468	1.16E-10	4.30E-10	AT1G26560.1 | Symbols: BGLU40 | beta glucosidase 40 | chr1:9178513-9181726FORWARD LENGTH=510
zhu1_Ghi#S42303905	1.172915697	1.65E-06	4.30E-10	AT1G80400.1 | Symbols: | RING/U-box superfamily protein |chr1:30225864-30227360 FORWARD LENGTH=407
zhu1_Ghi#S28685483	1.163164954	5.30E-13	4.30E-10	AT1G77380.1 | Symbols: AAP3, ATAAP3 | amino acid permease 3 |chr1:29075201-29077252 REVERSE LENGTH=476
zhu1_Ghi#S42324610	1.139958887	9.53E-06	4.30E-10	AT3G49810.1 | Symbols: | ARM repeat superfamily protein |chr3:18474936-18476282 REVERSE LENGTH=448
zhu1_Ghi#S42360646	1.132859494	5.45E-06	4.30E-10	AT5G02160.1 | Symbols: | unknown protein; FUNCTIONS IN:

				molecular_functionunknown; INVOLVED IN: biological_process unknown;LOCATED IN: chloroplast thylakoid membrane; EXPRESSEDIN: 23 plant structures; EXPRESSED DURING: 13 growthstages; Has 121 Blast hits to 121 proteins in 17species: Archae - 0; Bacteria - 0; Metazoa - 0; Fungi -0; Plants - 121; Viruses - 0; Other Eukaryotes - 0(source: NCBI BLink). | chr5:426392-427024 FORWARDLENGTH=129
zhu1_Ghi#S42291023	1.109679358	1.72E-13	4.30E-10	AT4G24990.1 | Symbols: ATGP4 | Ubiquitin family protein |chr4:12849973-12851249 REVERSE LENGTH=118
zhu1_Ghi#S28664540	1.102352958	5.83E-12	4.30E-10	AT5G11160.1 | Symbols: APT5 | adenine phosphoribosyltransferase 5 |chr5:3550774-3551986 FORWARD LENGTH=191
zhu1_Ghi#S28634130	1.09404353	3.86E-13	4.30E-10	AT5G43830.1 | Symbols: | Aluminium induced protein with YGL and LRDR motifs |chr5:17622593-17624239 REVERSE LENGTH=251
zhu1_Ghi#S42318858	1.092653172	2.57E-05	4.30E-10	AT1G65840.1 | Symbols: ATPAO4, PAO4 | polyamine oxidase 4 |chr1:24490173-24492728 FORWARD LENGTH=497
zhu1_Ghi#S42284305	1.085012179	6.44E-07	4.30E-10	AT3G19640.1 | Symbols: MGT4, MRS2-3 | magnesium transporter 4 |chr3:6820969-6823104 FORWARD LENGTH=484
zhu1_Ghi#S42335910	1.068092099	6.54E-06	4.30E-10	AT1G11720.2 | Symbols: SS3 | starch synthase 3 | chr1:3951597-3956840 FORWARDLENGTH=1094
zhu1_Ghi#S28633856	1.066593194	4.50E-06	4.30E-10	AT4G35300.4 | Symbols: TMT2 | tonoplast monosaccharide transporter2 |chr4:16796432-16799071 REVERSE LENGTH=739
zhu1_Ghi#S28678461	1.065884079	5.63E-11	4.30E-10	AT3G19640.1 | Symbols: MGT4, MRS2-3 | magnesium transporter 4 |chr3:6820969-6823104 FORWARD LENGTH=484
zhu1_Ghi#S42283189	1.065199959	3.09E-06	4.30E-10	AT5G49665.1 | Symbols: | Zinc finger (C3HC4-type RING finger) family protein |chr5:20167119-20169420 REVERSE LENGTH=740
zhu1_Ghi#S42294375	1.057599198	3.42E-13	4.30E-10	NoHits
zhu1_Ghi#S33793881	1.046693637	1.79E-07	4.30E-10	AT3G21360.1 | Symbols: | 2-oxoglutarate (2OG) and Fe(II)-dependent

				oxygenasesuperfamily protein | chr3:7522865-7524036 FORWARDLENGTH=330
zhu1_Ghi#S33810762	1.043366643	5.56E-05	4.30E-10	AT3G21510.1 | Symbols: AHP1 | histidine-containing phosphotransmitter 1 |chr3:7578432-7579537 REVERSE LENGTH=154
zhu1_Ghi#S42291268	1.04087063	1.23E-05	4.30E-10	AT1G60970.1 | Symbols: | SNARE-like superfamily protein |chr1:22448008-22449387 REVERSE LENGTH=177
zhu1_Ghi#S42362598	1.02706483	4.51E-06	4.30E-10	AT3G04090.1 | Symbols: SIP1;1, SIP1A | small and basic intrinsic protein 1A |chr3:1072340-1074031 REVERSE LENGTH=240
zhu1_Ghi#S30007407	1.02272154	0	4.30E-10	AT4G16260.1 | Symbols: | Glycosyl hydrolase superfamily protein |chr4:9200180-9201441 REVERSE LENGTH=344
zhu1_Ghi#S28680014	1.014991998	5.08E-06	4.30E-10	AT3G51520.1 | Symbols: | diacylglycerol acyltransferase family |chr3:19110739-19112518 FORWARD LENGTH=314
zhu1_Ghi#S42284281	1.010945165	2.60E-08	4.30E-10	AT5G46750.1 | Symbols: AGD9 | ARF-GAP domain 9 | chr5:18969950-18971817 REVERSELENGTH=402
zhu1_Ghi#S28642376	-1.008854744	7.79E-20	4.30E-10	AT4G17670.1 | Symbols: | Protein of unknown function (DUF581) |chr4:9833948-9834663 REVERSE LENGTH=159
zhu1_Ghi#S28670623	-1.016789459	8.41E-08	4.30E-10	AT1G50590.1 | Symbols: | RmlC-like cupins superfamily protein |chr1:18732378-18734053 REVERSE LENGTH=310
zhu1_Ghi#S42302947	-1.01971194	3.64E-36	4.30E-10	AT3G47730.1 | Symbols: ATATH1, ATH1, ABCA2 | ATP-binding cassette A2 |chr3:17594342-17598828 REVERSE LENGTH=983
zhu1_Ghi#S42287337	-1.020448631	7.48E-37	4.30E-10	AT2G15580.1 | Symbols: | RING/U-box superfamily protein | chr2:6797687-6798815FORWARD LENGTH=196
zhu1_Ghi#S28708850	-1.022688205	3.63E-33	4.30E-10	AT1G70170.1 | Symbols: MMP | matrix metalloproteinase | chr1:26424005-26425141FORWARD LENGTH=378
zhu1_Ghi#S28679896	-1.025023681	7.28E-07	4.30E-10	AT5G49520.1 | Symbols: WRKY48, ATWRKY48 | WRKY DNA-binding protein 48 |chr5:20090890-20092867 FORWARD LENGTH=399
zhu1_Ghi#S34535654	-1.028788404	4.34E-32	4.30E-10	AT1G80840.1 | Symbols: WRKY40, ATWRKY40 | WRKY DNA-binding protein 40

				|chr1:30383834-30385356 FORWARD LENGTH=302
zhu1_Ghi#S42291871	-1.034711367	5.46E-08	4.30E-10	NoHits
zhu1_Ghi#S33812841	-1.034862919	4.56E-06	4.30E-10	AT2G29500.1 | Symbols: | HSP20-like chaperones superfamily protein |chr2:12633279-12633740 REVERSE LENGTH=153
zhu1_Ghi#S42296316	-1.042837183	3.07E-13	4.30E-10	AT5G54770.1 | Symbols: THI1, TZ, THI4 | thiazole biosynthetic enzyme,chloroplast (ARA6) (THI1) (THI4) |chr5:22246634-22247891 FORWARD LENGTH=349
zhu1_Ghi#S28674764	-1.049556451	4.69E-06	4.30E-10	NoHits
zhu1_Ghi#S42303993	-1.052662362	4.04E-05	4.30E-10	AT1G50650.1 | Symbols: | Stigma-specific Stig1 family protein |chr1:18763773-18764297 REVERSE LENGTH=174
zhu1_Ghi#S42324594	-1.058775486	1.93E-55	4.30E-10	NoHits
zhu1_Ghi#S28674386	-1.058795906	1.25E-10	4.30E-10	AT2G46400.1 | Symbols: WRKY46, ATWRKY46 | WRKY DNA-binding protein 46 |chr2:19043676-19044754 REVERSE LENGTH=295
zhu1_Ghi#S33785628	-1.068092403	2.96E-05	4.30E-10	AT4G33040.1 | Symbols: | Thioredoxin superfamily protein |chr4:15940779-15941213 REVERSE LENGTH=144
zhu1_Ghi#S28691495	-1.076439746	2.36E-12	4.30E-10	AT5G15780.1 | Symbols: | Pollen Ole e 1 allergen and extensin family protein |chr5:5144898-5146297 REVERSE LENGTH=401
zhu1_Ghi#S28670636	-1.083359159	2.16E-05	4.30E-10	AT1G22470.1 | Symbols: | unknown protein; BEST Arabidopsis thaliana proteinmatch is: unknown protein (TAIR:AT1G72240.1); Has 1693Blast hits to 236 proteins in 54 species: Archae - 0;Bacteria - 8; Metazoa - 451; Fungi - 116; Plants - 94;Viruses - 2; Other Eukaryotes - 1022 (source: NCBIBLink). | chr1:7932908-7933348 REVERSE LENGTH=146
zhu1_Ghi#S28675029	-1.084580526	2.10E-17	4.30E-10	AT1G14870.1 | Symbols: PCR2 | PLANT CADMIUM RESISTANCE 2 | chr1:5128591-5129458REVERSE LENGTH=152
zhu1_Ghi#S42302358	-1.09029212	6.97E-07	4.30E-10	NoHits
zhu1_Ghi#S33790886	-1.09077166	9.54E-06	4.30E-10	AT5G49760.1 | Symbols: | Leucine-rich repeat protein kinase family

				protein |chr5:20216679-20221052 FORWARD LENGTH=953
zhu1_Ghi#S28642634	-1.092350337	8.26E-20	4.30E-10	AT2G40750.1 | Symbols: WRKY54, ATWRKY54 | WRKY DNA-binding protein 54 |chr2:17000636-17002354 REVERSE LENGTH=346
zhu1_Ghi#S28674900	-1.097921947	2.78E-22	4.30E-10	AT1G66880.1 | Symbols: | Protein kinase superfamily protein |chr1:24946928-24955438 FORWARD LENGTH=1296
zhu1_Ghi#S28642615	-1.099312456	3.79E-08	4.30E-10	AT1G14870.1 | Symbols: PCR2 | PLANT CADMIUM RESISTANCE 2 | chr1:5128591-5129458REVERSE LENGTH=152
zhu1_Ghi#S33796588	-1.10039203	4.15E-63	4.30E-10	NoHits
zhu1_Ghi#S33803441	-1.104846845	6.94E-06	4.30E-10	AT3G51325.1 | Symbols: | RING/U-box superfamily protein |chr3:19052259-19052531 FORWARD LENGTH=90
zhu1_Ghi#S42282492	-1.118786036	9.97E-11	4.30E-10	AT1G78700.1 | Symbols: BEH4 | BES1/BZR1 homolog 4 | chr1:29599854-29601539FORWARD LENGTH=325
zhu1_Ghi#S28679948	-1.120161123	1.95E-13	4.30E-10	AT5G26330.1 | Symbols: | Cupredoxin superfamily protein | chr5:9241614-9242635REVERSE LENGTH=187
zhu1_Ghi#S42314147	-1.127170797	4.04E-24	4.30E-10	AT2G33770.1 | Symbols: UBC24, ATUBC24, PHO2 | phosphate 2 |chr2:14277785-14281482 REVERSE LENGTH=907
zhu1_Ghi#S42276811	-1.133399842	2.37E-08	4.30E-10	NoHits
zhu1_Ghi#S33824436	-1.155939998	1.78E-14	4.30E-10	NoHits
zhu1_Ghi#S39759484	-1.163696101	0	4.30E-10	NoHits
zhu1_Ghi#S28705090	-1.184439275	1.13E-05	4.30E-10	AT1G65890.1 | Symbols: AAE12 | acyl activating enzyme 12 | chr1:24512598-24514611REVERSE LENGTH=578
zhu1_Ghi#S42323087	-1.188441206	5.84E-22	4.30E-10	AT3G14610.1 | Symbols: CYP72A7 | cytochrome P450, family 72, subfamily A,polypeptide 7 | chr3:4912565-4914503 FORWARD LENGTH=512
zhu1_Ghi#S30011418	-1.191601997	5.09E-146	4.30E-10	AT1G28280.2 | Symbols: | VQ motif-containing protein | chr1:9886297-9887395REVERSE LENGTH=243
zhu1_Ghi#S33804996	-1.193133378	1.65E-44	4.30E-10	AT3G28510.1 | Symbols: | P-loop containing nucleoside triphosphate

				hydrolasessuperfamily protein | chr3:10685656-10687248 FORWARDLENGTH=530
zhu1_Ghi#S33825092	-1.199139205	2.55E-20	4.30E-10	NoHits
zhu1_Ghi#S28643979	-1.223216092	0	4.30E-10	NoHits
zhu1_Ghi#S30016112	-1.227299981	2.67E-20	4.30E-10	NoHits
zhu1_Ghi#S42293757	-1.241606683	2.49E-20	4.30E-10	AT5G49610.1 | Symbols: | F-box family protein | chr5:20131448-20132527 REVERSELENGTH=359
zhu1_Ghi#S42360778	-1.244237192	6.41E-20	4.30E-10	AT1G29340.1 | Symbols: PUB17, ATPUB17 | plant U-box 17 | chr1:10264412-10266601FORWARD LENGTH=729
zhu1_Ghi#S28675946	-1.249266398	3.39E-07	4.30E-10	AT5G61820.1 | Symbols: | FUNCTIONS IN: molecular_function unknown; INVOLVEDIN: biological_process unknown; LOCATED IN: vacuole;EXPRESSED IN: 24 plant structures; EXPRESSED DURING: 15growth stages; CONTAINS InterPro DOMAIN/s: Stressup-regulated Nod 19 (InterPro:IPR011692); Has 30201Blast hits to 17322 proteins in 780 species: Archae -12; Bacteria - 1396; Metazoa - 17338; Fungi - 3422;Plants - 5037; Viruses - 0; Other Eukaryotes - 2996(source: NCBI BLink). | chr5:24834505-24836223 REVERSELENGTH=475
zhu1_Ghi#S33817364	-1.250469145	3.72E-31	4.30E-10	AT1G49570.1 | Symbols: | Peroxidase superfamily protein |chr1:18347077-18348712 FORWARD LENGTH=350
zhu1_Ghi#S33816319	-1.253043089	6.50E-06	4.30E-10	AT3G44350.2 | Symbols: anac061, NAC061 | NAC domain containing protein 61 |chr3:16022836-16024487 REVERSE LENGTH=241
zhu1_Ghi#S28675015	-1.253043089	1.09E-08	4.30E-10	AT2G17220.2 | Symbols: | Protein kinase superfamily protein |chr2:7487866-7489768 REVERSE LENGTH=413
zhu1_Ghi#S33796319	-1.264166467	1.49E-16	4.30E-10	AT3G47780.1 | Symbols: ATATH6, ATH6 | ABC2 homolog 6 | chr3:17624500-17628972FORWARD LENGTH=935
zhu1_Ghi#S42304308	-1.269552176	1.44E-07	4.30E-10	NoHits

zhu1_Ghi#S28694760	-1.27549794	1.33E-11	4.30E-10	AT3G54040.1 | Symbols: | PAR1 protein | chr3:20014032-20015299 REVERSELENGTH=183
zhu1_Ghi#S33807838	-1.280507972	2.94E-15	4.30E-10	AT2G45760.1 | Symbols: BAP2, BAL | BON association protein 2 |chr2:18847125-18847748 REVERSE LENGTH=207
zhu1_Ghi#S33822352	-1.286595649	5.70E-16	4.30E-10	AT4G27740.1 | Symbols: | Yippee family putative zinc-binding protein |chr4:13839148-13839616 FORWARD LENGTH=105
zhu1_Ghi#S33806012	-1.286750619	5.87E-31	4.30E-10	AT1G01490.2 | Symbols: | Heavy metal transport/detoxification superfamilyprotein | chr1:180401-182066 REVERSE LENGTH=177
zhu1_Ghi#S29394665	-1.298421084	6.63E-13	4.30E-10	AT5G48290.1 | Symbols: | Heavy metal transport/detoxification superfamilyprotein | chr5:19568980-19569658 FORWARD LENGTH=181
zhu1_Ghi#S42359955	-1.304386039	5.16E-23	4.30E-10	AT2G46400.1 | Symbols: WRKY46, ATWRKY46 | WRKY DNA-binding protein 46 |chr2:19043676-19044754 REVERSE LENGTH=295
zhu1_Ghi#S28644704	-1.313607766	0	4.30E-10	AT3G51430.1 | Symbols: YLS2, SSL5 | Calcium-dependent phosphotriesterasesuperfamily protein | chr3:19086548-19087909 FORWARDLENGTH=371
zhu1_Ghi#S28674653	-1.321389559	9.05E-16	4.30E-10	AT2G45760.1 | Symbols: BAP2, BAL | BON association protein 2 |chr2:18847125-18847748 REVERSE LENGTH=207
zhu1_Ghi#S28673288	-1.328415824	1.81E-06	4.30E-10	AT3G09270.1 | Symbols: ATGSTU8, GSTU8 | glutathione S-transferase TAU 8 |chr3:2848407-2849226 REVERSE LENGTH=224
zhu1_Ghi#S30006882	-1.329326674	4.39E-15	4.30E-10	AT5G52740.1 | Symbols: | Copper transport protein family |chr5:21382530-21383180 FORWARD LENGTH=118
zhu1_Ghi#S33816968	-1.338219634	2.01E-10	4.30E-10	NoHits
zhu1_Ghi#S33812971	-1.351446793	4.71E-06	4.30E-10	NoHits
zhu1_Ghi#S39759481	-1.37140586	0	4.30E-10	NoHits
zhu1_Ghi#S33826018	-1.372809572	7.56E-218	4.30E-10	AT3G11810.1 | Symbols: | unknown protein; BEST Arabidopsis thaliana proteinmatch is: unknown protein (TAIR:AT2G03330.1); Has 141Blast hits

				to 141 proteins in 21 species: Archae - 0;Bacteria - 9; Metazoa - 0; Fungi - 0; Plants - 131;Viruses - 0; Other Eukaryotes - 1 (source: NCBI BLink).| chr3:3727819-3728865 FORWARD LENGTH=348
zhu1_Ghi#S28671078	-1.376344667	1.79E-54	4.30E-10	AT2G15220.1 | Symbols: | Plant basic secretory protein (BSP) family protein |chr2:6608689-6609366 FORWARD LENGTH=225
zhu1_Ghi#S42317713	-1.413507761	3.33E-11	4.30E-10	AT3G63380.1 | Symbols: | ATPase E1-E2 type family protein / haloaciddehalogenase-like hydrolase family protein |chr3:23407112-23410213 REVERSE LENGTH=1033
zhu1_Ghi#S33840038	-1.417237264	3.77E-12	4.30E-10	AT3G22142.1 | Symbols: | Bifunctional inhibitor/lipid-transfer protein/seedstorage 2S albumin superfamily protein |chr3:7803604-7808046 REVERSE LENGTH=1480
zhu1_Ghi#S28675845	-1.419318254	1.25E-110	4.30E-10	AT4G39830.1 | Symbols: | Cupredoxin superfamily protein | chr4:18479103-18481184FORWARD LENGTH=582
zhu1_Ghi#S42280641	-1.427554329	0	4.30E-10	AT1G60420.1 | Symbols: | DC1 domain-containing protein | chr1:22261978-22264243FORWARD LENGTH=578
zhu1_Ghi#S33782966	-1.443825904	6.40E-12	4.30E-10	AT3G13130.1 | Symbols: | unknown protein; FUNCTIONS IN: molecular_functionunknown; INVOLVED IN: biological_process unknown;LOCATED IN: endomembrane system; EXPRESSED IN: malegametophyte; Has 140 Blast hits to 132 proteins in 41species: Archae - 2; Bacteria - 4; Metazoa - 29; Fungi -20; Plants - 51; Viruses - 0; Other Eukaryotes - 34(source: NCBI BLink). | chr3:4223008-4223613 FORWARDLENGTH=201
zhu1_Ghi#S42291130	-1.452239361	1.70E-234	4.30E-10	AT5G17540.1 | Symbols: | HXXXD-type acyl-transferase family protein |chr5:5782061-5783682 REVERSE LENGTH=461
zhu1_Ghi#S28676876	-1.517596306	1.33E-26	4.30E-10	AT5G40990.1 | Symbols: GLIP1 | GDSL lipase 1 | chr5:16418920-16420400 FORWARDLENGTH=374
zhu1_Ghi#S28656788	-1.525476193	9.93E-08	4.30E-10	AT5G55260.1 | Symbols: PPX2, PPX-2 | protein phosphatase X 2

				|chr5:22416808-22418621 FORWARD LENGTH=305
zhu1_Ghi#S42333464	-1.532792605	4.07E-128	4.30E-10	AT4G14680.1 | Symbols: APS3 | Pseudouridine synthase/archaeosinetransglycosylase-like family protein |chr4:8413443-8415311 REVERSE LENGTH=465
zhu1_Ghi#S28696727	-1.55789767	1.97E-09	4.30E-10	AT5G47850.1 | Symbols: CCR4 | CRINKLY4 related 4 | chr5:19378803-19381058 REVERSELENGTH=751
zhu1_Ghi#S33846154	-1.566694619	5.17E-26	4.30E-10	AT5G24530.1 | Symbols: DMR6 | 2-oxoglutarate (2OG) and Fe(II)-dependentoxygenase superfamily protein | chr5:8378964-8383154FORWARD LENGTH=341
zhu1_Ghi#S33784617	-1.578067255	0	4.30E-10	AT5G20230.1 | Symbols: ATBCB, BCB, SAG14 | blue-copper-binding protein |chr5:6826626-6827408 FORWARD LENGTH=196
zhu1_Ghi#S28670457	-1.580051962	2.75E-183	4.30E-10	AT3G56400.1 | Symbols: WRKY70, ATWRKY70 | WRKY DNA-binding protein 70 |chr3:20909082-20910409 REVERSE LENGTH=294
zhu1_Ghi#S28671572	-1.581087298	1.56E-67	4.30E-10	AT5G64810.1 | Symbols: WRKY51, ATWRKY51 | WRKY DNA-binding protein 51 |chr5:25908415-25909687 FORWARD LENGTH=194
zhu1_Ghi#S28676234	-1.586466823	1.01E-24	4.30E-10	NoHits
zhu1_Ghi#S42288583	-1.590355621	1.11E-69	4.30E-10	AT4G39330.1 | Symbols: ATCAD9, CAD9 | cinnamyl alcohol dehydrogenase 9 |chr4:18291268-18292772 FORWARD LENGTH=360
zhu1_Ghi#S33847052	-1.597426035	4.48E-07	4.30E-10	AT2G41180.1 | Symbols: | VQ motif-containing protein | chr2:17165242-17165667FORWARD LENGTH=141
zhu1_Ghi#S42306157	-1.599717846	1.00E-33	4.30E-10	AT4G34138.1 | Symbols: UGT73B1 | UDP-glucosyl transferase 73B1 |chr4:16348267-16349858 REVERSE LENGTH=488
zhu1_Ghi#S28644399	-1.617194854	2.50E-36	4.30E-10	AT4G21700.1 | Symbols: | Protein of unknown function (DUF2921) |chr4:11529956-11532844 FORWARD LENGTH=962
zhu1_Ghi#S33810112	-1.617747526	1.40E-45	4.30E-10	AT5G54490.1 | Symbols: PBP1 | pinoid-binding protein 1 | chr5:22121458-22121841FORWARD LENGTH=127

zhu1_Ghi#S42328484	-1.629377059	1.35E-27	4.30E-10	AT1G01490.2 | Symbols: | Heavy metal transport/detoxification superfamilyprotein | chr1:180401-182066 REVERSE LENGTH=177
zhu1_Ghi#S33803678	-1.632595038	4.52E-53	4.30E-10	AT1G19640.1 | Symbols: JMT | jasmonic acid carboxyl methyltransferase |chr1:6789166-6791708 REVERSE LENGTH=389
zhu1_Ghi#S33805995	-1.635900182	6.89E-06	4.30E-10	AT4G13395.1 | Symbols: DVL10, RTFL12 | ROTUNDIFOLIA like 12 |chr4:7785883-7786050 REVERSE LENGTH=55
zhu1_Ghi#S33799972	-1.635900182	1.44E-16	4.30E-10	AT5G45520.1 | Symbols: | Leucine-rich repeat (LRR) family protein |chr5:18449509-18453012 REVERSE LENGTH=1167
zhu1_Ghi#S42315404	-1.638068019	2.82E-10	4.30E-10	AT3G60220.1 | Symbols: ATL4, TL4 | TOXICOS EN LEVADURA 4 |chr3:22254790-22255794 REVERSE LENGTH=334
zhu1_Ghi#S28671549	-1.651007075	3.37E-05	4.30E-10	NoHits
zhu1_Ghi#S33814007	-1.673374888	1.32E-19	4.30E-10	AT5G26170.1 | Symbols: WRKY50, ATWRKY50 | WRKY DNA-binding protein 50 |chr5:9147176-9148128 REVERSE LENGTH=173
zhu1_Ghi#S28671034	-1.674047316	5.20E-89	4.30E-10	AT5G25250.1 | Symbols: | SPFH/Band 7/PHB domain-containing membrane-associatedprotein family | chr5:8749774-8751430 FORWARD LENGTH=470
zhu1_Ghi#S42302985	-1.692970368	1.16E-148	4.30E-10	AT5G57580.1 | Symbols: | Calmodulin-binding protein | chr5:23314994-23317683REVERSE LENGTH=647
zhu1_Ghi#S28645041	-1.698760206	6.33E-05	4.30E-10	AT2G15220.1 | Symbols: | Plant basic secretory protein (BSP) family protein |chr2:6608689-6609366 FORWARD LENGTH=225
zhu1_Ghi#S29994235	-1.705651291	1.60E-20	4.30E-10	AT4G38780.1 | Symbols: | Pre-mRNA-processing-splicing factor |chr4:18101438-18111029 REVERSE LENGTH=2332
zhu1_Ghi#S33803261	-1.730919563	3.07E-86	4.30E-10	AT2G29420.1 | Symbols: ATGSTU7, GST25, GSTU7 | glutathione S-transferase tau 7| chr2:12618111-12618871 REVERSE LENGTH=227
zhu1_Ghi#S42325367	-1.752870803	3.58E-40	4.30E-10	AT4G22200.1 | Symbols: AKT2/3, AKT3, AKT2, KT2/3 | potassium transport 2/3 |chr4:11746666-11750091 REVERSE LENGTH=802

zhu1_Ghi#S45786034	-1.785703588	4.45E-73	4.30E-10	AT2G31880.1 | Symbols: SOBIR1, EVR | Leucine-rich repeat protein kinase familyprotein | chr2:13554920-13556845 FORWARD LENGTH=641
zhu1_Ghi#S42297319	-1.791909175	2.36E-18	4.30E-10	AT5G66900.1 | Symbols: | Disease resistance protein (CC-NBS-LRR class) family |chr5:26714931-26717757 REVERSE LENGTH=809
zhu1_Ghi#S42300351	-1.847404288	2.63E-26	4.30E-10	NoHits
zhu1_Ghi#S28677246	-1.86046044	1.95E-08	4.30E-10	NoHits
zhu1_Ghi#S30015851	-1.873399496	3.44E-12	4.30E-10	AT2G18670.1 | Symbols: | RING/U-box superfamily protein | chr2:8093469-8094452FORWARD LENGTH=181
zhu1_Ghi#S33806908	-1.876719854	9.00E-24	4.30E-10	NoHits
zhu1_Ghi#S28642358	-1.903033157	2.82E-15	4.30E-10	AT3G49930.1 | Symbols: | C2H2 and C2HC zinc fingers superfamily protein |chr3:18510246-18510893 FORWARD LENGTH=215
zhu1_Ghi#S28674399	-1.926285076	1.12E-22	4.30E-10	NoHits
zhu1_Ghi#S42331422	-1.933764572	5.34E-45	4.30E-10	AT5G66590.1 | Symbols: | CAP (Cysteine-rich secretory proteins, Antigen 5, andPathogenesis-related 1 protein) superfamily protein |chr5:26574255-26574812 FORWARD LENGTH=185
zhu1_Ghi#S42315219	-1.936409294	5.08E-21	4.30E-10	AT3G56400.1 | Symbols: WRKY70, ATWRKY70 | WRKY DNA-binding protein 70 |chr3:20909082-20910409 REVERSE LENGTH=294
zhu1_Ghi#S42355978	-1.961282397	9.58E-164	4.30E-10	AT3G50770.1 | Symbols: CML41 | calmodulin-like 41 | chr3:18873987-18874604FORWARD LENGTH=205
zhu1_Ghi#S33817225	-1.966480148	2.77E-24	4.30E-10	AT1G09240.1 | Symbols: NAS3, ATNAS3 | nicotianamine synthase 3 |chr1:2984950-2985912 FORWARD LENGTH=320
zhu1_Ghi#S28686653	-2.006882502	1.84E-10	4.30E-10	AT5G66850.1 | Symbols: MAPKKK5 | mitogen-activated protein kinase kinase kinase 5| chr5:26695965-26699159 REVERSE LENGTH=716
zhu1_Ghi#S28636992	-2.063132979	3.31E-30	4.30E-10	AT3G54420.1 | Symbols: ATEP3, ATCHITIV, CHIV, EP3 | homolog of carrot EP3-3chitinase | chr3:20145935-20147034 FORWARD LENGTH=273
zhu1_Ghi#S28670485	-2.114767864	2.62E-61	4.30E-10	AT4G19950.1 | Symbols: | unknown protein; BEST Arabidopsis thaliana

				proteinmatch is: unknown protein (TAIR:AT5G44860.1); Has 338Blast hits to 330 proteins in 72 species: Archae - 2;Bacteria - 94; Metazoa - 7; Fungi - 0; Plants - 232;Viruses - 0; Other Eukaryotes - 3 (source: NCBI BLink).| chr4:10809977-10810942 FORWARD LENGTH=321
zhu1_Ghi#S28674059	-2.193381947	1.60E-30	4.30E-10	AT3G09270.1 | Symbols: ATGSTU8, GSTU8 | glutathione S-transferase TAU 8 |chr3:2848407-2849226 REVERSE LENGTH=224
zhu1_Ghi#S33804973	-2.235969576	4.41E-10	4.30E-10	AT4G19840.1 | Symbols: ATPP2-A1, ATPP2A-1, PP2-A1 | phloem protein 2-A1 |chr4:10774336-10775701 FORWARD LENGTH=246
zhu1_Ghi#S30007330	-2.237979367	1.51E-156	4.30E-10	NoHits
zhu1_Ghi#S33784215	-2.314626519	1.75E-93	4.30E-10	AT3G54420.1 | Symbols: ATEP3, ATCHITIV, CHIV, EP3 | homolog of carrot EP3-3chitinase | chr3:20145935-20147034 FORWARD LENGTH=273
zhu1_Ghi#S33822350	-2.330487174	8.23E-12	4.30E-10	NoHits
zhu1_Ghi#S33804884	-2.342437963	6.13E-115	4.30E-10	NoHits
zhu1_Ghi#S33808979	-2.359958293	3.82E-10	4.30E-10	NoHits
zhu1_Ghi#S28671517	-2.365252593	1.50E-28	4.30E-10	AT3G56400.1 | Symbols: WRKY70, ATWRKY70 | WRKY DNA-binding protein 70 |chr3:20909082-20910409 REVERSE LENGTH=294
zhu1_Ghi#S42310242	-2.621462799	3.90E-35	4.30E-10	AT4G31950.1 | Symbols: CYP82C3 | cytochrome P450, family 82, subfamily C,polypeptide 3 | chr4:15455163-15457090 FORWARDLENGTH=512
zhu1_Ghi#S42319364	-4.481381189	0	4.30E-10	AT4G31940.1 | Symbols: CYP82C4 | cytochrome P450, family 82, subfamily C,polypeptide 4 | chr4:15452040-15453966 FORWARDLENGTH=524
zhu1_Ghi#S28692004	-14.35294343	6.19E-29	4.30E-10	AT4G21410.1 | Symbols: CRK29 | cysteine-rich RLK (RECEPTOR-like protein kinase)29 | chr4:11402463-11405025 REVERSE LENGTH=679

Supplementary Table 2 Primers used in this study. The primers of qRT-PCR are marked by the 'RL', The primers of RT-PCR are marked by the 'RT'.

Gene name	Forward primer	Reverse primer
SSN-RL	CTTGGGTTACAACTATGCCATGT	ATTGAGCCTTCTATTTCCGAGAC
SSN1-RL	GACCAACATTAAATCTACCCCGCT	ACCGTAAGAACCAAAATGCTAAACC
SSN2-RL	GACTAACATTAAATCTACACCGCTTGA	ATTGTTCTTCACACCTGATTATTGCT
SSN3-RL	ATGGTTTGATTGACTTGACTGGGAC	GCTTGATTACTGGAATCTTCAACTGG
SSNi	GGGGACAATTTGTACAAAAAAGCAGGCTGCAGTGTTGTTGCAGAGGAAGACCAGA	GGGGACCACTTTGTACAAGAAAGCTGGGTGACACCAATTTGCCGATGTCTGTTT
SSN1oe	GGGGACAAGTTTGTACAAAAAAGCAGGCTGCATCTCTACACTTTCTTAACACAGCAG	GGGGACCACTTTGTACAAGAAAGCTGGGTGTTCTGTCCTTCACACTTTATTATTGC
SSN2oe	GGGGACAAGTTTGTACAAAAAAGCAGGCTGCCAACAACTTCTTCAGAAGATCTCTGCA	GGGGACCACTTTGTACAAGAAAGCTGGGTCTTGTTCTTCACACCTGATTATTGCT
SSN3	ATGGATCTTCTTGATCTCTCTATTTTTGGC	ATGCTTGATTACTGGAATCTTCAACTGG
UTR1i	GGGGACAAGTTTGTACAAAAAAGCAGGCTGCGATTGCAATAATAAAGTGTGAAGGAC	GGGGACCACTTTGTACAAGAAAGCTGGGTGTTTAAAAAGAAATGGAATATATTTTGC
UTR2i	GGGGACAAGTTTGTACAAAAAAGCAGGCTGCATTAAAATTTAGATGAAGATAGCAA	GGGGACCACTTTGTACAAGAAAGCTGGGTGTCTCTAACAAATATTTGTTTATTTATTA
UTR3i	GGGGACAAGTTTGTACAAAAAAGCAGGCTGCAATCCAGTTGAAGATTCCAGTAATC	GGGGACCACTTTGTACAAGAAAGCTGGGTGAAGCAACACTCAAATCTAGATGTTTTA
pSSN1	GGGGACAAGTTTGTACAAAAAAGCAGGCTCGTTCAATTAATTTTTTTAAGGGTCTTGT	GGGGACCACTTTGTACAAGAAAGCTGGGTCCACTGCTGTGTTAAGAAAGTGTAGAG
pSSN2	GGGGACAAGTTTGTACAAAAAAGCAGGCTCGACTCAATCACAGTCCCTGGTATG	GGGGACCACTTTGTACAAGAAAGCTGGGTCCTCTGCTTATTTTGATGGGAAAGT
SSN1-GFP	GGGGACAAGTTTGTACAAAAAAGCAGGCTTCATGGATCTTCTTGATTTCTCCACT	GGGGACCACTTTGTACAAGAAAGCTGGGTTGTTATAGAGCTCAGGAGCAAGGC
PR1-RL	GACTCTGTCCATCTTGGTTGTGCTA	TTTAGTAAGGTTTTTGACCGACGAA
PR2-RL	CCACCAGCAGCAGAAGTTATCG	TTCAAGGTTTGCACTCGGAAGA
PR3-RL	ACTCCACAATCACCGAAGCCAT	GCATTCCAACCCTTACCACATTC
PR5-RL	GCCGTGATTCATACAGTTATCCTCA	TTGGCTCTTACTTCCGACCATCT
PR10-RL	TTTTACACTGTTGGCGACTATGTGA	TTAGTTGCAGGCATCAGGGTTAG
NPR1-RL	GCGAATCGTTGCTTTCTTCTTCA	CACGTGGTGCTGTTGTTGTTACTG
ICS1-RL	ATGGATGAATGGGTGCGAAGG	AAGAATGCCAGAGGTAAGAGGAGGA

EDS1-RL	GCAGCAACAGCTCCTCTACCTCAA	GGCAGACCAAGACGCTACAGATACA
PAD4-RL	GGATGGAAGAATGGAAAGAAATGAA	GAACTAGGAAAGCAGACTAAGGAACCA
CAT-RL	TGTTCCCTATCCCACCTGCTGT	GAATCGCTCTTGCCTGTCTGC
PR1-RT	CTTGTGCACTCCAGTGGACCTTAT	CCATTCAGTAGGCAAATCAGTACATA
PR5-RT	CCTCCTCTTCTTTAGCGTCCTTTTC	GAGCTCTTAACTCATTCGGGCATT
LOX1-RL	TAGAGAGGACATTTTGCCCTGG	GGTCAAGGTCGTCCAGAGATTTTA
LOX2-RL	TTTTACTCCTACTGTGCTTCCCTTCA	CCATGCCAACTCCGATTTTTCTC
LOX5-RL	CAATACGCTTACGGAGGCTACTTC	AGACCTTCTCAGGGTTCTTTTCAA
GhLOX1	GATGGACTGGAGATTTGGTTTGC	GGAGTTCTTTCCACCAGGCTTGA
AOS-RL	CGATGTCCCAACTTCAATCTCAAAC	ATCCGACGGTGGAGAATAAACAGT
AOC4-RL	AATAGAGCATAAACCCGAAATGAAAG	CAAAAATGCCAGACCCACCAGTA
JAZ1-RL	GACCAAATCTTGTGGCATCTACCTC	CTTTGGTTCCACTGCTGCTGATT
JAZ3-RL	GCGGGTGAAGTGAATGTCTTTGAT	GAACTGGTTTCAAAATAGGAGTCTGGA
JAZ10-RL	TGAATAATTAAGGACGGGGTGTTC	ATCAGACCGTCGAGACAAGCATTA
AOS-RT	GACAAAGCCCTTGCATTGACGA	CCGTTGATGAAATCGGTGGTGT
JAZ1-RT	AGTTTTCAAACATCAGAAGGGAGGA	TCCGCGGGAAAATCATTATATACA
JAZ3-RT	GCCAAGCCCTTTACCTGTTTCATC	AAACCAAAATGCCAAACATAAACCT
SAD-RL	ATCAGATGGATTTCACGAGCAAGT	AAGGAGTAAGGCTAGCACCTGTTTC
FAD2-RL	CACTGTTTCCAACGCTCACTTATC	AAGATAGTGGCTGAGGGAGGTTG
FAD3-RL	GCTCTACGGTGTTCCCTACTTGATT	CGGTCTATTGTTGTAAGCCCTCCT
FAD6-RL	ACAGGCACCATGCAAAGACAAAC	GCTATGGACATCCAAGGTCGAAA
FAD7-RL	ACCCAAGCCAACAAGAATAGCAG	GTGGAACAGAAGTCCAGGAAAGAGT
FAD8-RL	ATGGAGAATTAGCCACAGGACTCA	AACCGCAATGTTCGTGTTAGAGTAT
α-DOX1-RL	CACTTTGGGAGGTTTAGTTGGTCT	TGTATGGAATCTGGTAAAAGTTGGTG
α-DOX2-RL	TCATTTGGAGGACACTCAACAGGTA	GTGTCCTTGTATTGAGAAAGCCAAA
HPL-RL	CATTGAGCAGCACGGTAAAGAAG	CCGAAGGCATTAAACCCAAGAGT
AZI1-RL	AAACCCTACTCCCTCCCCATCT	AGACCTTGGATGAGAGAGCAGCA
AZI1-RT	AAACCCTACTCCCTCCCCATCT	CGACATTGGAAGCCAGAGGGTA
V-LOX1	CGGGGTACCATGCAGACTCGAGAAGAGTTGATCG	CGCGGATCCCGTTGTTCATCTCTACAATCCGTTCC
V-LOX2	CGGGGTACCCCGAAGAGGCAACTGAAAGAGAT	CGCGGATCCGCTCAGATGGAGATGCTATATGGAA
UBQ7	GAAGGCATTCCACCTGACCAAC	CTTGACCTTCTTCTTCTTGTGCTTG
35S	AAGTTCATTTCATTTGGAGAGGACAC	
NPT II	CGGCGATACCGTAAAGCACGAGGAA	CGACGTTGTCACTGAAGCGGGAAGG
